# Organization and Dynamics of Focal Adhesions: Light Diffraction Analysis of Cellular Adhesion on Nanopatterned Surfaces

**DOI:** 10.1021/acsami.6c04698

**Published:** 2026-06-10

**Authors:** Inna Szekacs, Szabolcs Novák, Boglarka Kovacs, Zoltán Dicső, Beatrix Péter, Attila Bonyár, Roman Popov, Andreas Frutiger, Robert Horvath

**Affiliations:** † Nanobiosensorics Laboratory, Institute of Technical Physics and Materials Science, Centre for Energy Research, HUN-REN, Konkoly-Thege Miklós Street 29-33, 1121 Budapest, Hungary; ‡ Department of Electronics Technology, Faculty of Electrical Engineering and Informatics, 309392 Budapest University of Technology and Economics, Egry J. Street 18., 1111 Budapest, Hungary; § Department of Biological Physics, ELTE Eötvös Loránd University, 1117 Budapest, Hungary; ∥ Lino Biotech AG, Soodstrasse 52, 8134 Adliswil, Switzerland; ⊥ Institute of Biophysics, Biological Research Centre HUN-REN, 6726 Szeged, Hungary

**Keywords:** diffraction-based optical sensing, nanophotonic biosensor, label-free, live-cell adhesion, cell−substrate interactions

## Abstract

This study presents the first application of nanophotonic sensing modality for investigating live-cell adhesion, introducing a novel label-free optical method to monitor specific nanoscale structural changes at the cell–substrate interface. Our method utilizes receptor molecules immobilized in a diffraction pattern with a precise submicron periodicity, which provides superior sensing volume confinement through the spatial lock-in amplification principle. This internal nanoscale ruler enables the investigation of otherwise diffraction-limited phenomena with higher specificity and reduced background noise, ultimately providing more insight into nanoscale adhesion organization dynamics. To complement this approach, resonant waveguide grating (RWG) biosensing and holographic microscopy were used to characterize adhesion behavior and morphological changes of HeLa cells on RGD-functionalized substrates. The nanophotonic readout revealed distinct multistep adhesion dynamics associated with integrin clustering, nanoscale redistribution of adhesion-associated molecular assemblies, and focal adhesion remodeling. Quantitative analysis estimated that approximately 1.22 × 10^6^ RGD-specific integrins contributed to the coherent adhesion signal per HeLa cell within 2 h of adhesion, while the measured redistribution dynamics corresponded to an effective velocity of approximately 0.09 μm/h. Enzymatic digestion of the glycocalyx with neuraminidase significantly altered adhesion behavior, highlighting the importance of membrane organization in integrin-mediated adhesion. Furthermore, histamine stimulation modulated adhesion dynamics and induced cytoskeleton-associated remodeling responses that depended on the prior adhesion time. Together, these results demonstrate the sensitivity of this diffraction-engineered sensing strategy to subtle mechanobiologically regulated changes in the cellular microenvironment and establish its potential as a powerful tool for real-time studies of the biophysical regulation of cell adhesion and receptor-mediated signaling.

## Graphical abstract

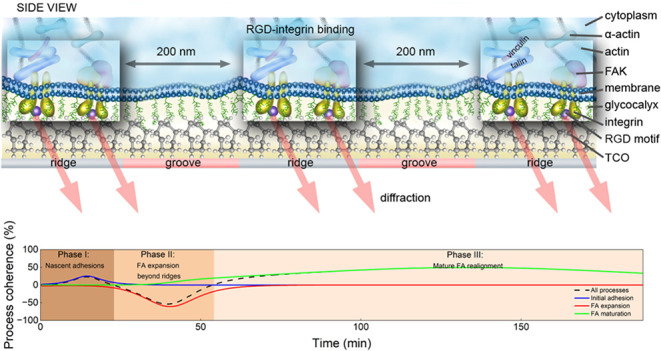

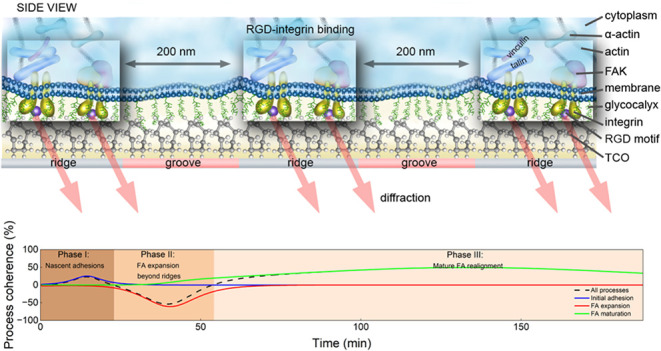

## Introduction

Cell adhesion is a highly regulated and dynamic process that governs essential biological functions, ranging from embryonic development and tissue morphogenesis to wound healing and immune surveillance. Understanding the molecular mechanisms underlying cell adhesion is fundamental to deciphering key aspects of cellular behavior, including proliferation, differentiation, survival, apoptosis, and the regulation of cellular senescence.^1,2^ The interactions between cells and the extracellular matrix (ECM) serve as critical modulators of mechanotransduction and play an important role in the initiation and maintenance of cellular senescence by transducing biochemical and biophysical cues.^3^

Beyond its physiological significance, aberrant adhesion mechanisms contribute to pathological conditions, including cancer metastasis, fibrosis, and inflammatory diseases. Dysregulated adhesion facilitates tumor progression by promoting invasion, immune evasion, and metastatic dissemination.^4^ Investigating cell adhesion at molecular and biophysical levels is therefore crucial not only for advancing fundamental cell biology but also for developing novel therapeutic strategies in regenerative medicine, oncology, and biomaterial engineering.

At the molecular level, cell adhesion is primarily mediated by integrin receptors, a family of transmembrane proteins composed of α- and β-subunits. Integrins serve as bidirectional biomechanical sensors, anchoring cells to the ECM while simultaneously transmitting and responding to both biochemical and physical cues from their surroundings.^5^ Their function is tightly regulated through conformational changes, clustering, and interactions with intracellular partners, such as talin and kindlin–processes that modulate integrin activation and ligand-binding affinity. This mechanochemical regulation enables integrins to finely detect variations in ECM rigidity, ligand composition, and spatial organization, ultimately orchestrating essential cellular behaviors such as migration, adhesion, and mechanotransduction–particularly in contexts like cancer.^5^

The Arg-Gly-Asp (RGD) tripeptide motif is a key sequence found in many ECM proteins like fibronectin, vitronectin, and fibrinogen. Artificial polymers incorporating the highly effective RGD motif are widely used to precisely enhance and control cell adhesion on synthetic surfaces. Most of the RGD-binding integrins, i.e., αvβ1, αvβ3, αvβ5, αvβ6, αvβ8, α5β1, αIIbβ3, and α8β1, are expressed at elevated levels in various cancer types.^4^

The glycocalyx is a dense layer of glycoproteins and glycolipids that covers the surface of most mammalian cells and plays a critical role in regulating cell–substrate interactions. The contribution of the glycocalyx to integrin-mediated cell adhesion remains a topic of active investigation. Studies propose that a bulky glycocalyx promotes integrin clustering by exerting steric pressure that drives receptors into localized high-density regions.^6,7^ This clustering is thought to enhance adhesion through cooperative binding, resulting in stronger and more stable interactions with ligands.^8^ Enzymatic modification of glycocalyx components can significantly alter integrin accessibility, clustering, and downstream signaling. Notably, Kanyo et al. demonstrated that different concentrations of the Chondroitinase ABC enzyme can elicit opposing effects on cell adhesion: low doses enhanced adhesion, possibly by unmasking integrin-binding domains, while high doses disrupted proteoglycan-mediated structural integrity, leading to reduced adhesion.^9^ The extended glycocalyx physically impedes close membrane–substrate contact, limiting integrin access to binding sites and thereby reducing adhesion efficiency. Chighizola et al. demonstrated that the glycocalyx modulates cell adhesion and mechanotransduction in a nanotopography-dependent manner.^10^ Using PC12 cells, they showed that even subtle variations in nanoscale topographical features can dramatically impact early adhesion behavior and force distribution at the membrane. Importantly, the effect of glycocalyx reduction varied across different nanostructures, indicating that both the glycocalyx and the ECM’s nanometric architecture act interdependently to regulate integrin activation, adhesion site formation, and cytoskeletal tension. These findings reinforce the idea that cells decode extracellular cues through an integrative mechanism involving glycocalyx configuration, integrin expression/activation, and actomyosin contractility. The evidence from enzymatic treatments and nanotopography studies underscores the glycocalyx’s decisive role in modulating adhesion dynamics, focal adhesion (FA) maturation, and mechanosensing in cancer cells. This has critical implications for understanding tumor cell behavior in complex microenvironments and designing surface-engineered biomaterials or therapies that exploit these glycan-mediated mechanisms.

To study cell adhesion processes, researchers have increasingly turned to optical biosensors, which offer several advantages over traditional techniques. These biosensors enable real-time, label-free monitoring of cell adhesion kinetics and offer high-throughput capabilities.^11^ Among the various optical biosensor techniques, resonant waveguide grating (RWG) biosensors have gained popularity due to their ability to detect changes in the local refractive index within a sensing volume of approximately 150 nm from the sensor surface.^12^ This technique enables real-time, high-throughput monitoring of cell adhesion kinetics, dynamic mass redistribution, and integrated response profiles by accurately detecting shifts in mass and refractive index as cells adhere, change morphology, or undergo biochemical alterations.^9,12–19^

Since refractometric optical biosensors rely on changes in the refractive index in the volume illuminated by the evanescent field to detect molecular interactions, any refractive index changes in this thin layer–caused by nonspecific binding of ligand molecules, variations in supernatant composition or ionic strength, or minor temperature fluctuations–affect the detected phase signal.

Focal molography (FM) is a label-free optical biosensing method based on the principle of coherent light scattering. As a novel optical diffractometric biosensor technique, FM has emerged as a promising approach for studying cellular processes like adhesion, offering enhanced molecular specificity and robustness in complex media–a critical requirement for obtaining physiologically relevant data.^20–24^ The core of the method involves analyzing how light, typically from a laser, scatters in a predictable manner from receptor molecules that have been arranged in a precise, periodic pattern on the sensor surface. This engineered molecular structure functions as a synthetic phase hologram or “mologram”.^25^

Like other evanescent-field biosensors, FM detects refractive-index changes within ∼150 nm of the sensor surface. The distinguishing feature is that the receptors are not distributed uniformly but are immobilized in a precisely engineered submicron periodic pattern—the mologram—which acts as a diffractive optical element. Only bound molecules whose lateral distribution reproduces this submicron periodicity scatter the guided light in phase and contribute to the focused diffraction signal. Thus, the intensity of the focal spot serves as a quantitative readout of the surface-bound interaction, offering high sensitivity, while any other refractive index changes in the evanescent field volume–such as that from nonspecifically absorbing proteins or bulk solution changes–is effectively suppressed. In essence, the method is self-referencing on the submicron scale, directly tackling the persistent challenge of background noise in label-free assays, suppressing noncoherent background signals. An early detailed theoretical foundation of FM was comprehensively described by Fattinger.^20^

The technique’s inherent robustness stems from the spatial lock-in amplification principle. This means the specific binding signal is encoded into the mologram’s high spatial frequency. In contrast, environmental noise sources such as temperature fluctuations, bulk refractive index changes, and nonspecific binding interferences are typically random or uniform across the surface, corresponding to a low spatial frequency. This clear difference in spatial characteristics allows the system to effectively isolate the desired molecular signal from environmental noise, enabling highly stable measurements even without active temperature control.^23,24^ This spatial lock-in, realized by the submicron periodic arrangement of receptor molecules, facilitates direct sampling of the signal in Fourier space through optical diffraction. Such an approach inherently performs reference subtraction–by focusing only on changes that match the mologram’s periodicity–and enhances the signal-to-noise ratio by isolating the desired signal from broadly distributed background scattering.

In this sense, we could consider the mologram as an internal nanoscale ruler that enables the investigation of diffraction-limited processes on the nanoscale in a label-free manner. Compared to traditional optical label-free biosensing methods that utilize evanescent fields to establish a sensing volume defined primarily in the vertical direction (such as, for example, Surface Plasmon Resonance (SPR),^26,27^ or Resonant Waveguide Grating (RWG)^13^), FM introduces a critical second dimension, as the diffraction pattern with precisely defined nanoscale periodicity constrains the sensing volume also laterally.^23,24,28,29^

Thanks to these principles FM offers several advantages compared to other techniques: (i) unparalleled sensing volume confinement: enabled by the submicron diffraction pattern (ii) higher specificity: FM focuses on particular receptor–ligand interactions, providing more targeted insights into molecular-level adhesion processes; (iii) reduced background noise: the submicron pattern arrangement helps to minimize nonspecific signals, improving the signal-to-noise ratio; (iv) compatibility with complex biological samples: FM can potentially be used with whole cells or even tissue samples, offering a more physiologically relevant context for adhesion studies.

FM has been applied in various biological contexts, including the *in situ* monitoring of antibody production in hybridoma cell cultures over extended periods,^30^ the characterization of intracellular signaling pathways in living cells,^31,32^ and the kinetic affinity determination and biomarker quantification in complex matrices.^33^

In this study, we present the first application of FM for live-cell adhesion analysis, highlighting specific early stage interactions between cells and functionalized surfaces. Cell adhesion kinetics were evaluated for different cell densities to RGD motifs. Based on the sensorgram data, we quantified the effective coherent contribution of RGD-specific integrins and estimated the redistribution dynamics of integrin-associated adhesion assemblies between grooves and ridges of the nanopatterned surface. We further investigated how enzymatic modification of the glycocalyx influences the early phases of HeLa cell adhesion and nanoscale adhesion organization on RGD-functionalized substrates. Additionally, we demonstrate that histamine-induced cellular response dynamics depend strongly on the duration and maturation state of cell adhesion.

## Results and Discussion

### Label-Free Optical Assays In Situ Monitor HeLa Cell Adhesion

The kinetics of HeLa cells’ spreading were monitored using a well-established RWG biosensor and the holography microscope HoloMonitor M4 over a period of up to 2 h in complete culture medium (Figure 1). Figure 1a provides an overview of the experimental design of RWG measurement, and Figure 1b shows typical sigmoid-like curves when HeLa cells were adhered to the RGD-motifs through integrins. The process of HeLa cells’ adhesion to RGD-containing surface occurs in several phases: (i) within the first few minutes, cells settle down and initial attachment appears; (ii) cell spreading, formation of adhesive structures takes place up to 60 min (depending on experimental conditions). As the number of cells increases, more cells attach to the biosensor surface, increasing the refractive index and enhancing the RWG signal. However, at high cell densities, the surface becomes fully occupied, leading to a plateau where further increases in cell number (e.g., from 21,000 to 42,000) do not change the signal (Figure 1b).

**Figure 1:**
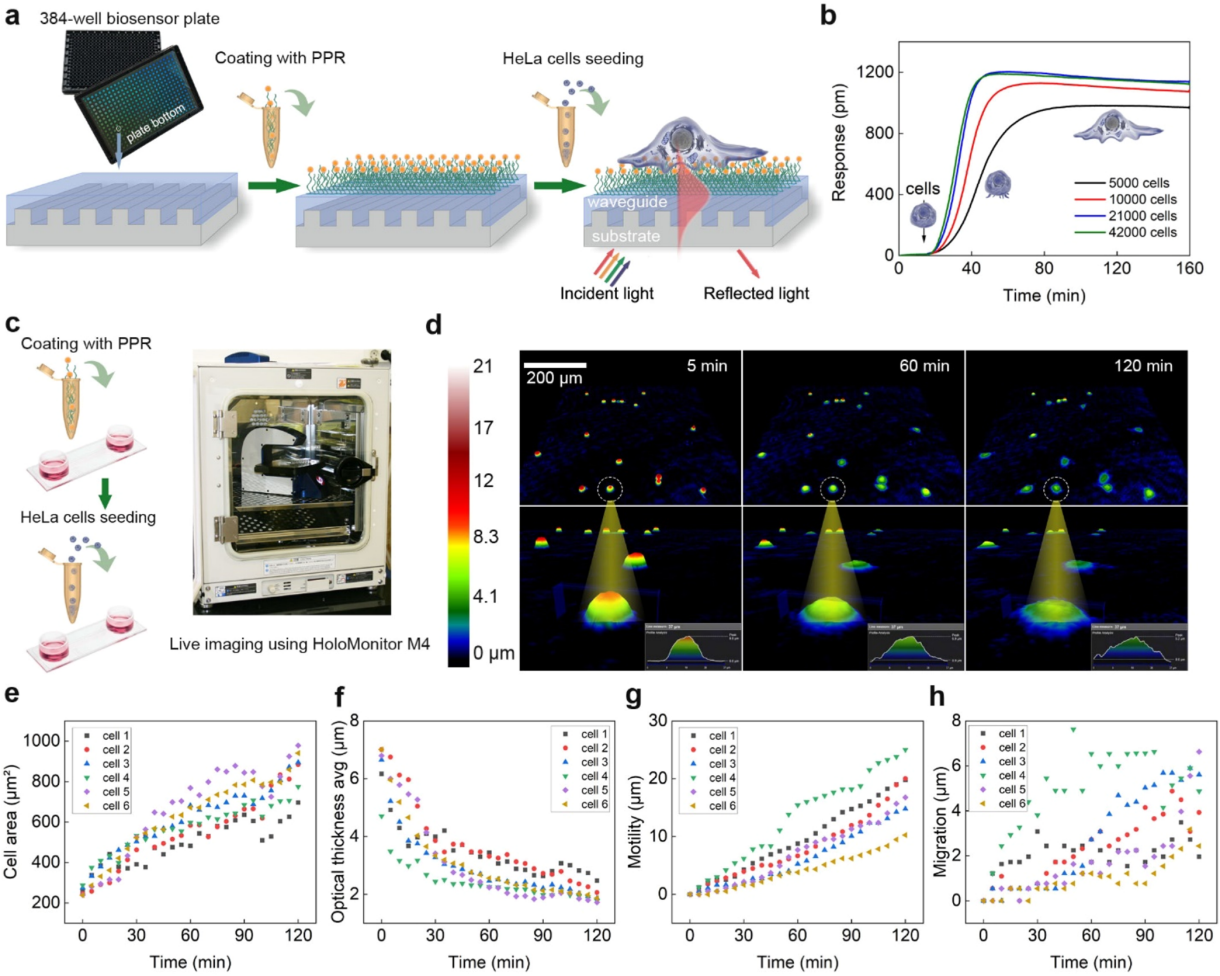
Overview of the workflow and HeLa cell adhesion kinetics with morphological changes sensed over a 2 h period in complete culture medium. (a) RWG sensors in the wells of the biosensor plate were functionalized with RGD motifs, and HeLa cells at different concentrations were then seeded onto the sensor surface. (b) The kinetics of cell adhesion to RGD motifs on the RWG sensor surface were recorded as a shift in the measured resonant wavelength. (c) Ibidi μ-Slide I was functionalized with RGD motifs, and HeLa cells were plated at a density of 100,000 cells per channel. Live images were captured every 5 min inside a humidified incubator using the HoloMonitor M4 instrument. (d) Representative 3D images captured with a holographic microscope at three time points (5, 60, and 120 min), showing the evolution of cell adhesion. The scale bar indicates the height distribution of cells using a color-coded scale from 0 to 21 μm. Cell morphological changes during the adhesion process are patterned as spreading area (e), averaged optical thickness (f), motility (g), and migration (h).

HeLa cells undergo dynamic morphological changes during the adhesion process, characterized by increased spreading area, decreased height, and variable motility and migration patterns. Figure 1c shows the experiment setup using the holographic microscope HoloMonitor M4, and Figure 1d–h present a detailed analysis of cell behavior over a 2 h period of HeLa cell adhesion. The cell area increases steadily over time, indicating progressive cell attachment and spreading (Figure 1e). The optical thickness shows an initial peak (6–7 μm) in the first few minutes, followed by a gradual decrease to around 2 μm by 120 min (Figure 1f). This suggests cell flattening during the adhesion process. Cell motility increases linearly over time, with different cells showing varying degrees of movement (Figure 1g). The migration patterns vary significantly between cells, with some cells showing more directed movement (reaching 6–7 μm displacement) while others display more limited migration (2–3 μm) (Figure 1h).

### Focal Molography Uncovers the Specificity of HeLa Cell Adhesion

With the RWG technique, we obtain an integrated response profile of cell adhesion, as all events occurring in the evanescent field contribute to the biosensor signal. In contrast, FM offers a more specific and targeted approach to studying cell adhesion processes, as it can selectively detect interactions at nanoscale-ordered molecular recognition sites.

In terms of nomenclature, the mologram is composed of two functionally distinct lines, referred to as the ridges and the grooves. One should not think of those as physical structures like trenches but rather as alternating regions defined by their chemical function and how they ultimately affect the interference of light in the focal spot. The ridges are the active regions chemically designed for receptor immobilization and subsequent specific binding, which lead to constructive interference. The grooves are passive, nonbinding regions, often coated with repellent molecules like PEG (poly­(ethylene glycol)), which contribute to destructive interference. One ridge and one groove constitute one period of the mologram. For example, a mologram composed of biotin ridges and PEG grooves is denoted as [Biotin | PEG].

The primary readout signal in FM is the coherent surface mass density (CMD, Γ_coh_). This value is precisely defined as the Fourier coefficient of the mass distribution, meaning it quantifies the exact component of the total bound mass that spatially matches the mologram’s repeating pattern. It is the only parameter that can be accurately quantified from the measured diffraction efficiency.^22^

It is crucial to distinguish the coherent mass density (CMD) from two broader quantities:

1. *Total diffractometric surface mass density* (DMD):
  This represents the overall surface mass that follows the affinity distribution defined by the lithographic pattern. It is always larger than the coherent mass density and is related to it through the **analyte efficiency** η_*A*_ ^22^ (eq 1).
1 $$\Gamma_{\mathrm{diff},\mathrm{tot}}=\frac{\Gamma_{\mathrm{coh}}}{\eta_{A}}$$
  The analyte efficiency describes what fraction of the total bound mass contributes coherently to the first-order diffraction signal. Because η_*A*_ depends on the details of the lithography, surface chemistry, and molecular size, it must either be measured or assumed for each chip.^25^
2. *The total refractometric mass density* (RMD):
  This quantity describes the total surface-mass-induced change in refractive index within the evanescent field. It includes *all* bound material, coherent or not, under constant assay conditions.

CMD therefore captures the amplitude of the periodic component of the surface mass that produces the diffracted light, whereas DMD and RMD quantify broader aspects of total mass accumulation. For an ideal sinusoidal modulation of surface mass, the total diffractometric mass density equals twice the coherent mass density, since only half of the accumulated mass contributes constructively to the diffraction signal.^22,34^ When one is interested in the absolute molecular surface densities required to produce a given CMD, DMD is the appropriate measure. However, it is not directly accessible from the optical signal and requires knowledge (or assumption) of the analyte efficiency.

Another important definition is the process coherence. A process coherence of 100% means that the measured DMD and RMD are equal. Therefore, all mass that is deposited in the evanescent field precisely follows the initial affinity distribution.

### Chemistry on the MoloChip

The principle of FM and instrument setup are illustrated in Figure 2. The sensor chip utilizes a single-mode optical waveguide integrated with a grating coupler. Light guided through the waveguide is diffracted by the mologram and focused into a diffraction-limited spot. A central curved recess within the mologram is designed to suppress second-order Bragg reflection (Figure 2a).^20^ The system features an open four-channel cuvette with dimensions of 8.1 mm × 0.9 mm × 2.5 mm (length × width × height), with a usable volume of 18 μL per channel (Figure 2b,d). Each channel contained nine square mologram spots, each with a size of 400 μm × 400 μm and a carrier period of the mologram of 406 nm. The carrier period is defined as the grating period that leads to perpendicular outcoupling of the guided mode.^35^ Due to focusing, the periods of the molographic lines are chirped in mode propagation direction and vary from 403 to 409 nm. The ridges and grooves are therefore roughly 200 nm in width. The focal distance of the molograms is 12 mm in air. Within these spots, the anchor-to-anchor distance was 4 nm, resulting in approximately 1 × 10^10^ binding sites per spot and 9 × 10^10^ binding sites per channel. The molography readout wavelength in the cited foundational studies was typically 632.8 nm (e.g., He–Ne laser as described in Frutiger et al.);^21^ however, in our current system, a readout wavelength of 785 nm is employed.

**Figure 2:**
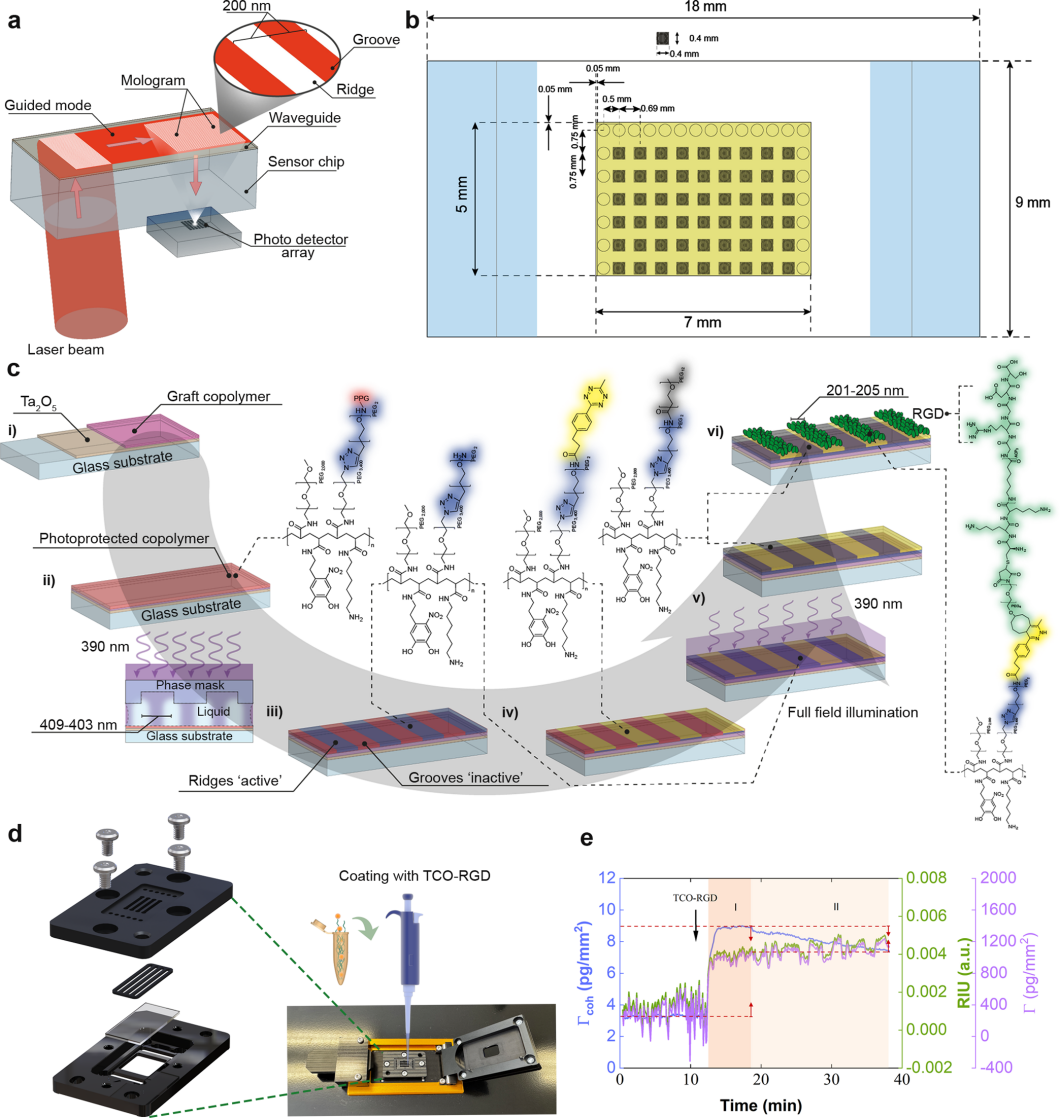
MoloChip functionalization and binding characterization. (a) Working principle of focal molography: The sensor chip consists of a single-mode optical waveguide coated with a graft copolymer layer. A grating coupler launches the guided light mode, which is diffracted by the structured arrangement of biomolecules forming the mologram. This diffraction generates a focal spot, where the light intensity scales quadratically with the number of analyte molecules bound to the mologram. Only specifically bound molecules contribute to the signal, while nonspecifically interacting components do not. The evolving light intensity at the focal spot is recorded in real time using a photodetector array. (b) Dimensions of the MoloChip. (c) Schematic illustration of MoloChip surface chemistry buildup: (i) A 145 nm thick metal oxide (Ta_2_O_5_) layer is coated with a thin photosensitive copolymer containing azido-terminated PEG3400 side chains. (ii) An alkyne-functionalized, photocaged amine is subsequently attached to the surface via copper-catalyzed azide–alkyne cycloaddition (“click chemistry”). This yields a monolithic polymer layer with grafted PEG3400 chains bearing a triazole linkage to a photocaged amine with the amine–triazole segment highlighted in blue and the photolabile protecting group (PPG) moiety in red. (iii) Spatially selective UV exposure is applied through a phase mask. The optical interference pattern generated by the mask results in nanostructures with half the period of the phase mask (409–403 nm), leading to feature sizes of 201–205 nm line width. Only the exposed regions (ridges) are activated by photolysis, releasing the protecting group and generating free amines (blue), while the unexposed grooves remain protected. This yields a nanopatterned surface with alternating functionalities: [NH_2_ | NH-photoprotected]. (iv) The ridges (activated amines) are functionalized with an amine-reactive Me-Tz derivative (yellow) to allow bioorthogonal reactivity via TCO-tetrazine chemistry. Resulting structure: [Me-Tz | NH-photoprotected]. (v) A uniform UV exposure is applied to cleave the remaining photoprotecting groups in the grooves, yielding free amines across the entire surface. The grooves are then functionalized with NHS-PEG_12_-Ome (gray) to passivate the nonbinding background and reduce nonspecific binding. Surface composition: [Me-Tz|PEG]. (vi) In the final step, the molographic binding sites (ridges) are click-reacted with TCO-RGD (TCO-PEG_4_-Maleimide-S-Cys-Lys-Lys-(Acp)_3_-GRGDS) via tetrazine-TCO ligation, introducing integrin-recognizing ligands selectively. The entire structure is highlighted in green, while the RGD motif is specifically indicated in red. This click conjugation step was performed in-house. Final pattern: [RGD|PEG], forming a spatially resolved and biofunctional molographic surface. (d) Design of MoloChip and holder. (e) Representative sensorgram showing TCO-RGD binding kinetics.

The MoloChip surface was functionalized with a two-dimensional polymer brush architecture based on poly­(ethylene glycol) (PEG3400) to ensure antifouling properties and enable bioorthogonal ligand immobilization. The underlying polymer matrix, as described in detail in Gatterdam et al.,^30^ features a defined side-chain composition comprising 15.0% azide functionalities, 42.5% primary amines, and 42.5% nitrocatechol anchors, facilitating robust surface grafting.

Azide-bearing side chains were selectively addressed via copper­(I)-catalyzed azide–alkyne cycloaddition (CuAAC), utilizing a bifunctional photolabile amine linker with a photoprotected amine group and an alkyne moiety for the click reaction. This reaction yielded a 1,4-disubstituted 1,2,3-triazole intermediate. Upon UV-mediated deprotection, the liberated amine was derivatized with NHS-activated methyltetrazine (Me-Tz), yielding surface-displayed Me-Tz motifs.

These reactive handles were subsequently engaged in an inverse electron-demand Diels–Alder cycloaddition with trans-cyclooctene (TCO)-modified ligands (e.g., RGD peptides), producing a stable methyl-dihydropyridazine adduct with concomitant nitrogen extrusion. Each derivatized PEG3400 chain thus harbors two orthogonal click-chemistry adducts culminating in site-specific ligand presentation.

Given the 15% azide content in the base polymer and a selective 25% substitution with Me-Tz groups, the final surface coverage of RGD corresponds to ∼3.75% of the total side chains. This yields a highly controlled ligand density, tailored for quantitative molographic biosensing applications.

To initiate cell adhesion, the surface of the 2D PEG-[Me-Tz|PEG] MoloChip was modified with RGD motifs through third generation click chemistry. Figure 2c shows a schematic illustration of the biomimetic surfaces created. A molographic pattern is composed of ridges and grooves that direct diffracted light into a diffraction-limited focal point. The ridges represent regions of higher refractive index, while the grooves between them have a lower refractive index. Reactive groups (Me-Tz) are sinusoidally distributed on the ridges of the mologram with an offset.^22,25^ The grooves consist of a small PEG moiety. Underneath both ridges and grooves, a brushed-copolymer coating^30^ resides to minimize nonspecific binding, although complete repellency may not always be achieved in a complex, serum-containing environment.^36,37^ However, molecules adsorbed nonspecifically (randomly) to the underlying PEG on the sensor surface do not contribute to the coherent signal in the mologram’s focal point.

The reaction between TCO-RGD and Me-Tz groups is considered extremely fast and irreversible under physiological conditions. Figure 2e demonstrates the molographic sensorgram depicting the interaction dynamics of TCO-RGD with Me-Tz-functionalized surfaces. Immediately after the addition of TCO-RGD, all three signals–the coherent mass density (CMD, Γ_coh_, blue), the refractometric mass density (RMD, Γ, purple), and the refractive index units (RIU, green)–exhibit a sharp rise reaching saturation in about 2 min. The RIU and the RMD signals show typical adsorption-like kinetics, indicating mass accumulation in the sensing volume. While CMD shows a plateau for approximately 6 min (Phase I), the CMD signal subsequently begins to decrease during Phase II. This suggests an ongoing reaction between TCO-RGD and Me-Tz in the grooves. This phenomenon may be explained by a backfilling effect, which minimizes refractive index differences between ridges and grooves. A similar effect was observed using a different measurement setup and chip type (see Supporting Information, Figure S1).

TCO-RGD was attached to the Me-Tz groups of ridges, with a CMD of around 6 pg/mm^2^ (Figure 2e, Phase I). Considering a molecular weight of 1657.9 g/mol and a peptide purity of 99%, this corresponds to an estimated surface coverage of 2.16 × 10^9^ molecules/mm^2^ (eq 2). Accordingly, the total number of molecules immobilized on the 160,000 μm^2^ (0.16 mm^2^) mologram coherently was approximately 3.45 × 10^8^.

2 $$N_{\mathrm{TCO}‐\mathrm{RGD},\mathrm{coh}}=\frac{\Gamma_{\mathrm{coh}\left(\mathrm{TCO}‐\mathrm{RGD}\right)}\cdot p\cdot N_{A}}{M_{\mathrm{TCO}‐\mathrm{RGD}}}$$

where: Γ_coh(TCO‑RGD)_ = 6 × 10^–12^ g/mm^2^, *p* = 0.99 (purity factor), *M*_TCO‑RGD_ = 1657.9 g/mol, and *N*_A_ = 6.022 × 10^23^ mol^–1^.

The excess TCO-RGD reacted in the grooves (Figure 2e, Phase II), reducing the CMD value to approximately 4 pg/mm^2^. This corresponds to an estimated surface coverage of 1.44 × 10^9^ molecules/mm^2^, with ∼2.30 × 10^8^ molecules immobilized on the mologram coherently.

While the theoretical maximum number of binding sites per mologram is around 10^10^ (the mean distance anchor to anchor is 4 nm, area per binding site is 16 nm^2^; the size of mologram is 160,000 μm^2^ = 1.6 × 10^11^ nm^2^), only ∼2.30 × 10^8^ RGD molecules were effectively immobilized coherently.

However, the total number of immobilized RGD molecules is higher because the analyte efficiency of the mologram—defined as the ratio of coherent to total diffractometric mass density—is below 100%.^25^ For the fabrication process used here, this efficiency is typically in the range of 10–20%, depending on the phase-shift mask design and the illumination dose applied during surface patterning. When this factor is considered, together with the fact that the CMD was calculated using the average refractive index increment for proteins (rather than the specific value for the RGD–TCO molecule), the theoretical and experimental values agree well. Using an analyte efficiency of **10%** (as specified by the manufacturer), the corrected total number of immobilized RGD molecules on the mologram is estimated to be approximately ∼2.30 × 10^9^.^25^

To describe the spatial distribution, the total diffractometric surface mass density can be modeled as a sinusoidal modulation superimposed on a constant background to yield an analyte efficiency of 10%

3 $$\Gamma \left(x\right)=\Gamma_{0}\left[1+A\sin\left(x\right)\right]$$

where Γ_0_ is the mean mass density and *A* = 0.2 is the relative amplitude (corresponding to a 40% peak-to-peak variation between ridges and grooves). Integrating the modulated term (*A* sin­(*x*)) over half periods shows that approximately 82% of the modulated mass resides on the ridges and 18% on the grooves. When the constant background is included, this yields the overall molecular distribution, with 56.4% of the RGD molecules on the ridges and 43.6% in the grooves, corresponding to about 1.3 × 10^9^ and 1.0 × 10^9^ molecules, respectively.

### Comprehensive Analysis of HeLa Cell Adhesion to RGD-Functionalized Surfaces Using FM Data

Molographic measurements of HeLa cells’ adhesion to the RGD-functionalized surface revealed multiphase nanoscale organizational dynamics associated with integrin engagement and adhesion maturation (Figure 3a). Immediately after cell introduction (Phase I), the CMD (blue), RIU (green), and RMD (purple) signals rise sharply, consistent with rapid integrin-mediated attachment (see Figure 3f for schematics). The RMD and the RIU signals exhibit adsorption-like kinetics, reflecting mass buildup of cells and ECM within the sensing volume. Due to the sinusoidal distribution of RGD on ridges and grooves, integrins initially bind preferentially to ridge regions of slightly higher ligand density and more favorable topography for membrane contact.

**Figure 3:**
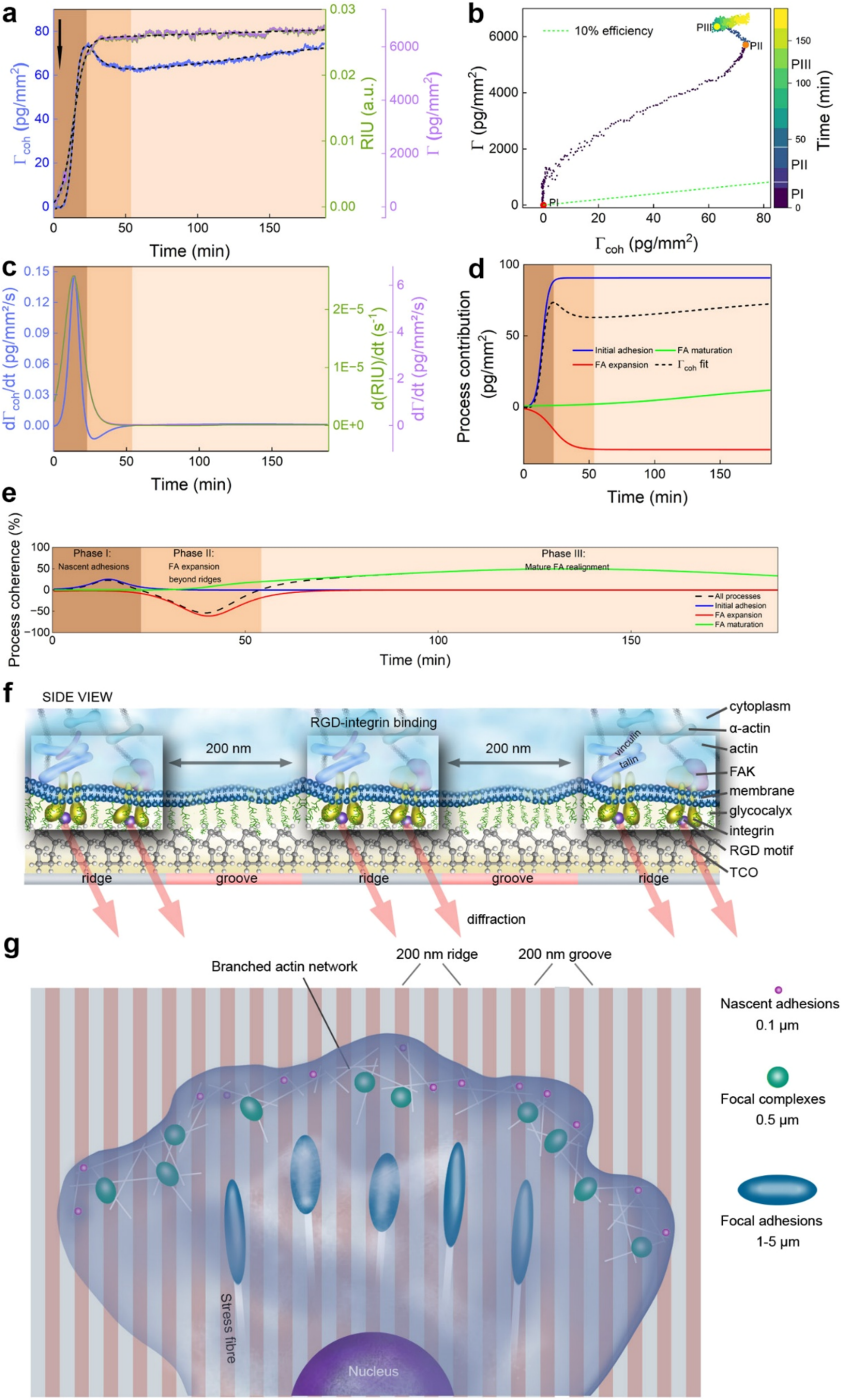
HeLa cell adhesion to RGD motifs on the MoloChip. (a) Representative sensorgram of the measurement in DMEM (CMD – blue, RIU – green, and RMD – purple). The black arrow indicates the time point at which 30,000 HeLa cells were added per channel. The measured signal reflects the collective response of adherent cells within the sensing area (approximately 267 fully adherent cells per mologram, 160,000 μm^2^). Solid lines represent experimental data while dashed lines show fitted models (4PL with linear drift for RMD, *R* ^2^ = 0.996; 4PL with sigmoid transitions for CMD, *R* ^2^ = 0.998). Background shading indicates three distinct phases: Phase I (0–23 min), Phase II (23–54 min), and Phase III (54–192 min). (b) Scatter plot of CMD versus RMD showing the trajectory of focal adhesion development. Points are colored by time using a viridis colormap (purple to yellow, 0–192 min). Phase start points are indicated by colored markers (PI–PIII) while boundaries are indicated by white lines on the colormap. The green dashed line represents the expected 10% analyte efficiency if all the material in the evanescent field were organized in the same spatial distribution as the original RGD pattern. The deviation from this line reveals changes in molecular organization relative to the nanopattern. (c) Time derivatives of CMD (blue, left axis) and RMD (purple, right axis) showing the rates of mass accumulation. The derivative analysis reveals the kinetics of each process, with phase transitions occurring at zero crossings of the derivative ratio. (d) CMD signal decomposition into individual processes. The black dashed line shows the total CMD fit; colored lines represent initial adhesion (blue), FA expansion (red), and FA maturation (green). (e) Process coherence relative to the RGD nanopattern. The black dashed line shows overall coherence; colored lines indicate contributions from individual processes. The horizontal dashed line at 100% marks perfect pattern fidelity. (f) Schematic representation of the molographic signal arising from the superposition of scattered electric fields generated by spatially organized integrin-associated molecular assemblies on the waveguide surface. (g) Schematic illustration of the cell adhesion maturation on the mologram, progressing from nascent adhesions to focal adhesion structures.

Interestingly, a decrease in the CMD signal is observed after approximately 23 min (Figure 3a, Phase II), while the RMD and the RIU signals have not yet reached saturation. This suggests ongoing integrin flow from the cells, with newly available integrins interacting with RGD motifs in the grooves (Figure 3g), thereby flattening the optical modulation pattern (backfilling effect). Because the RMD and RIU signals continue increasing during Phase II, the CMD decrease is unlikely to reflect net mass loss from the evanescent field. Instead, the data are more consistent with nanoscale spatial redistribution and reorganization of adhesion-associated material that reduces the sinusoidal refractive index modulation while overall surface-associated mass continues to accumulate.

Later (Phase III), CMD partially recovers as integrin-associated adhesions become more spatially organized during maturation. This recovery may reflect preferential reclustering toward ridge regions with favorable ligand presentation and topography, but may additionally arise from force-guided alignment and anisotropic organization of mature adhesions along cytoskeletal stress directions. Such stress-dependent spatial ordering would be fully consistent with the observed increase in coherent diffraction signal because FM is sensitive to collective nanoscale organization and spatial coherence. Since stress fiber orientation and traction anisotropy were not directly measured in the current experiments, we cannot definitively distinguish between ridge-guided reorganization and stress-guided alignment processes, and both mechanisms are therefore considered plausible contributors to the observed CMD recovery. Although the difference in RGD concentration between ridges and grooves is relatively small (∼10%), integrins may still preferentially bind to RGD motifs on the ridges due to a combination of chemical and topographical cues. The nanotopography of the surface not only subtly modulates ligand density but also influences the geometry of membrane contact and focal adhesion assembly. Ridges may present a more accessible and flatter substrate for initial integrin engagement, thereby promoting membrane spreading and enhancing mechanotransduction.^38^ These physical cues, in concert with localized increases in RGD density, likely promote integrin clustering and more stable adhesion on ridge regions.^39,40^ Integrins undergo force-dependent clustering and turnover during adhesion maturation, increasing local mass density (Figure 3a, Phase III). Although these processes are mechanically regulated, the present measurements do not directly resolve local traction forces or molecular tension states. Instead, force-dependent adhesion remodeling is inferred indirectly through changes in nanoscale spatial organization detected by the coherent diffraction signal. The spatial density of RGD motifs significantly impacts focal adhesion formation and stability. A critical RGD spacing for Hela cell adhesion has been reported to be ∼ 50 nm, while larger spacing >100 nm results in a weak adhesion.^13^

Importantly, the same RMD values associated with different CMD data (Phase III in Figure 3a) (or vice versa) clearly indicates structural rearrangement of cell adhesion molecules on the molograms (Figure 3b). Basically, in this case, the same amount of material is present in the evanescent field, but with different 2D spatial organization, resulting in distinctly different diffraction capability and thus different coherent mass signals. Of note, quantitatively, the maximum RMD reached ∼6360 pg/mm^2^. Assuming an analyte efficiency of 10% in coherent detection, the expected CMD contribution would be ∼640 pg/mm^2^. This implies that only ∼10% of the deposited mass directly contributes to specific RGD–integrin diffraction signals, while ∼90% originates from nonspecific cellular material drawn into the evanescent field (e.g., cytosolic volume or membrane components).

Taken together, the sensorgram shown in Figure 3a reveals three distinct phases of adhesion: (I) rapid initial integrin engagement with the RGD motifs, predominantly on ridge regions of the nanopatterned surface, yielding a strong coherent signal; (II) a transient decrease in CMD consistent with lateral expansion and nanoscale redistribution of adhesion-associated assemblies across both ridges and grooves, thereby reducing sinusoidal refractive index modulation; and (III) partial increased spatial organization of mature adhesions, potentially arising from preferential ridge-associated reclustering, force-guided alignment, or anisotropic organization along cytoskeletal stress directions. These dynamics were well described by 4-parameter logistic (4PL) and sigmoid functions achieved R^2^ values exceeding 0.99 for both total RMD and CMD signals (dashed lines show fitted models in Figure 3a). The total RMD increased from a baseline of approximately – 100 pg/mm^2^ to a maximum of ∼6360 pg/mm^2^, illustrating the overall magnitude of mass accumulation within the evanescent field. The linearly modeled baseline drift was relatively small (∼0.03 pg/mm^2^/s). For CMD, the initial 4PL rise reached ∼90 pg/mm^2^, consistent with early integrin engagement and nanoscale clustering during Phase I. The subsequent negative sigmoid (Phase II) decreased CMD by ∼30 pg/mm^2^, consistent with lateral expansion and spatial redistribution of adhesion-associated assemblies across neighboring groove regions, resulting in partial loss of coherence. Partial recovery in Phase III contributed ∼15 pg/mm^2^ to CMD, likely reflects increasing spatial organization of mature adhesions through ridge-associated reclustering and/or force-guided alignment processes. The timing of these events is captured by the fitted inflection points, with the negative sigmoid centered at ∼1323 s and the positive sigmoid at ∼8000 s, corresponding to the approximate onsets of Phase II decay and Phase III recovery, respectively. A full list of all fitted parameters, including asymptotes, slopes, and transition widths, is provided in Table 4.

Fitting of the CMD signal allowed us to calculate its time derivative d­(CMD)/dt, which revealed three distinct phases separated by zero crossings at 22.5 and 53.75 min (Figure 3c), thus precisely indicating the points of phase transitions. For reference, the derivative of the RMD fit, d­(RMD)/dt, is also shown in the figure to contextualize the changes in coherent mass relative to total mass accumulation. These phases are consistent with fundamentally different molecular processes occurring at the nanopatterned surface.

The model fitted to the CMD signal could be decomposed into three distinct mathematical components (Figure 3d): an initial 4PL rise, followed by two sequential sigmoidal terms. While purely phenomenological in nature, these components are likely to represent underlying biological processes occurring at the RGD nanopatterned surface. The initial 4PL rise (blue curve) can be attributed to rapid integrin engagement at ridge sites and the formation of nascent adhesions. The first sigmoid contribution (red curve) is consistent with lateral expansion and nanoscale redistribution of these early adhesion assemblies into larger focal adhesion structures spanning neighboring grooves, thereby disrupting the sinusoidal refractive index modulation. Finally, the second sigmoid (green curve) likely reflects increasing spatial organization and maturation of focal adhesions, in which integrins and associated proteins undergo ridge-associated reclustering and/or force-guided anisotropic alignment that partially restore the coherent signal. Together, these results indicate that the observed CMD dynamics are not uniform but rather consist of multiple overlapping processes, each leaving a characteristic signature in the optical readout.

Process coherence analysis (Figure 3e) quantifies how closely mass accumulation follows the lithographically defined RGD nanopattern present on the surface. The pattern can be approximately described by a sinusoid with a constant offset, yet the actual distribution is more elaborate.^25^ Coherence is defined as the ratio of CMD to RMD derivatives normalized by analyte efficiency, with 100% representing perfect fidelity to the underlying molographic pattern. Thus, any value below 100% means that mass is pulled uniformly into the evanescent field, independent of the underlying receptor distribution and shall be a general tool to analyze and report cellular molography measurements. The overall coherence trace (black dashed line) revealed three distinct regimes aligned with the phases of adhesion.

During Phase I (0–23 min), early nascent adhesion structures (100–150 nm) likely formed predominantly at RGD-rich ridge sites. The rise in CMD to approximately 90 pg/mm^2^ is consistent with early integrin engagement, nanoscale clustering (∼20–60 molecules per cluster), accompanied by the recruitment of talin and kindlin. These early adhesions are short-lived (∼1–2 min) and relatively incoherent due to concurrent nonspecific membrane contact. Correspondingly, coherence remained low throughout this phase, averaging 12.9% and rarely exceeding ∼25%. This low coherence indicates that most material entering the evanescent field, such as membrane protrusions, cytosolic components, and unstructured adhesion proteins, was not spatially aligned with the underlying RGD nanopattern, while only a minor fraction contributed to the ridge-localized coherent adhesion structures.

In Phase II (23–54 min), these early adhesions likely evolved into larger adhesion assemblies, including focal complexes (200–400 nm) and early focal adhesions (1–5 μm). As these structures expanded across neighboring ridges and grooves, increasing engagement of groove-associated RGD motifs reduced the sinusoidal refractive index modulation. The subsequent decrease in CMD by ∼30 pg/mm^2^ is therefore consistent with lateral expansion and nanoscale redistribution of adhesion-associated assemblies beyond the ridge-confined regions. This “flattening” of the optical pattern reduces diffraction efficiency despite continued mass accumulation within the evanescent field. In parallel, coherence transiently dropped below zero, indicating that the evolving spatial organization of adhesion-associated material actively opposed the original sinusoidal refractive index modulation generated by the molographic pattern.

During the final phase, Phase III (54–175 min), CMD partially recovers by ∼15 pg/mm^2^, likely reflecting increased spatial organization of mature adhesion structures along the topographical cues. This recovery may arise from force-dependent recruitment of proteins such as zyxin and α-actinin, as well as from enhanced coupling between integrin-based adhesions and the actin cytoskeleton, which together may contribute to increased spatial clustering toward RGD-rich ridge regions. Concurrently, coherence partially recovered to ∼40% as adhesion-associated assemblies exhibited increased alignment with the ridge-guided topography and/or anisotropic organization along cytoskeletal stress directions. However, the signal never reached full pattern fidelity, underscoring that only a subset of the total accumulated mass remained coherently organized according to the original RGD nanopattern.

Importantly, these optical signatures likely arise from multiple overlapping processes occurring simultaneously, including adhesion assembly/disassembly, integrin redistribution, cytoskeletal coupling, molecular recruitment, and force-dependent structural remodeling, which cannot be fully deconvolved in the current measurements.

HeLa cell adhesion thus follows the classical sequence of adhesion maturation: formation of transient nascent adhesions (∼tens of seconds to 2 min lifetime), progressing to focal complexes (∼5–10 min), and eventually maturing into larger focal adhesions (>20 min).^41^ While nascent adhesions can form independently of actomyosin tension, their stabilization and maturation require vinculin, FAK, and linkage to actin filaments. Actomyosin contractility subsequently drives the growth of focal adhesions, stabilizing them under force and reducing integrin mobility. Quantitative live-cell analyses report focal adhesion assembly and disassembly rate constants of ∼0.03 min^–1^ and ∼0.02 min^–1^, respectively.^42^

Importantly, multiple adhesion types may coexist and interconvert within a single cell.^43^ Canonical adhesions typically progress from nascent adhesions to focal adhesions under β1 integrin engagement, whereas αvβ5 integrins can mediate reticular adhesions at flat clathrin lattices, particularly under vitronectin substrates or reduced β1 activity.^44^ These adhesion modes often coexist, sharing components and dynamically exchanging with one another.

The open four-channel cuvette and integrated mologram identifier (Figure 4a) allow imaging of adhered cells directly after the measurement. Figure 4b shows representative images captured before and after the washing steps in a cell adhesion experiment, demonstrating that the cells adhere specifically to the mologram regions while nonadherent cells can be effectively removed. Each channel contains nine square mologram spots. Figure 4c presents a magnified view of a single mologram with adhered HeLa cells. Figure 4d shows vinculin staining in HeLa cells adhered to RGD-functionalized MoloChip after 2 h-adhesion.

**Figure 4:**
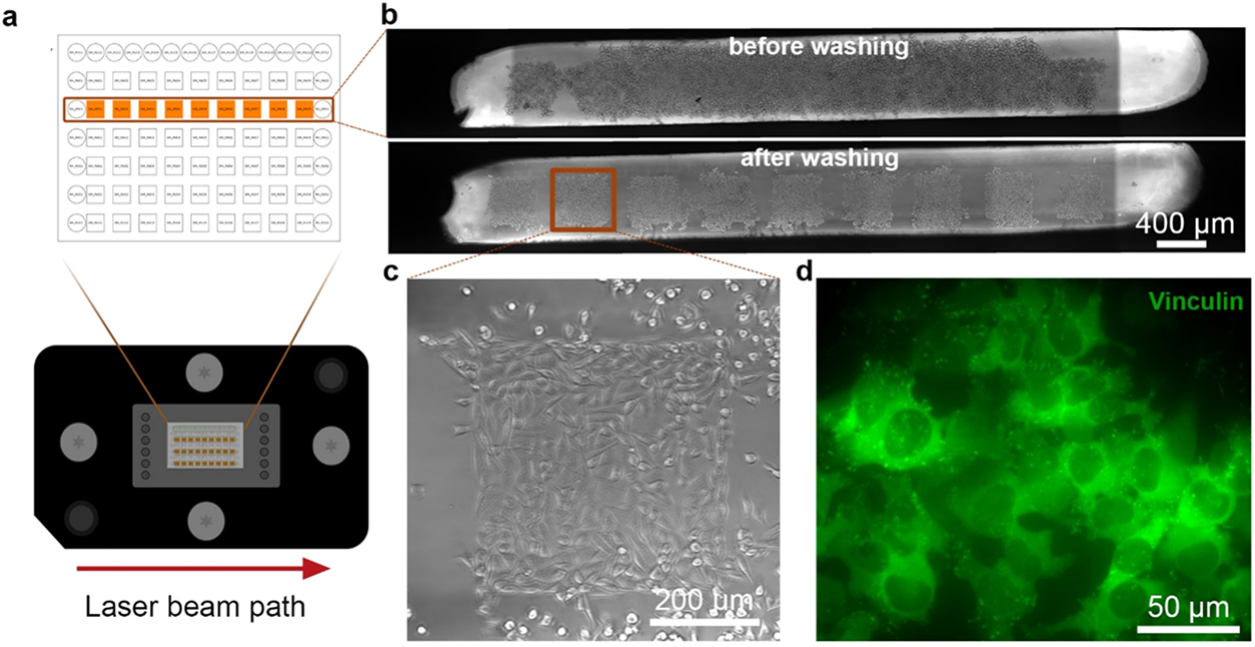
Cell adhesion visualization on the MoloChip. (a) Schematic illustration of the chip holder with a 4-channel setup and mologram identifier. (b) One channel of the MoloChip containing nine molograms, shown before (including nonadherent cells) and after washing. (c) Single mologram displaying adhered HeLa cells. (d) Vinculin staining in HeLa cells adhered to RGD motifs on the MoloChip.

### Quantification of RGD-Specific Integrins Based on Measured CMD

We determined from the microscopy images that a single HeLa cell covers an average surface area of approximately 600 μm^2^ on the mologram’s surface after 2 h of adhesion. Based on the known mologram area of 400 μm × 400 μm (160,000 μm^2^), this corresponds to a theoretical maximum of ∼267 fully adherent cells per mologram under conditions of complete surface coverage.

The main RGD-binding integrins in HeLa are αvβ3, αvβ5, and α5β1.^45^ Their molecular weight typically ranges between 200–300 kDa, depending on the specific combination of subunits. Based on the measured CMD signal, an approximate density of coherently organized integrin-associated molecules contributing to the molographic signal can be estimated (eq 4). For this calculation, an average integrin molecular weight of 250 kDa (250,000 g/mol) is assumed. In Phase I, corresponding to a CMD of 75 pg/mm^2^, the calculated coherent integrin-equivalent density was approximately 181 integrins/μm^2^. However, after correcting for the analyte efficiency (assumed to be 10%), the actual number of RGD-associated integrin molecules contributing to the adhesion process was estimated to be around 1810 integrins/μm^2^. Given a mologram area of 160,000 μm^2^, this corresponds to a total of 2.89 × 10^8^ integrins per mologram. For a typical HeLa cell with a surface area of 600 μm^2^, the estimated number of RGD-associated integrins was 1.08 × 10^6^ per cell (Table 1).

4 $$N_{\mathrm{integrins}}=\frac{\Gamma_{\mathrm{coh}\left(\mathrm{integrins}\right)}\cdot N_{A}\cdot S}{\eta_{A}\cdot M_{\mathrm{integrin}}}$$

where Γ_coh(integrins)_ represents measured CMD in g/μm^2^, *M*_integrin_ = 250,000 g/mol, *N*_A_ = 6.022 × 10^23^ mol^–1^, *S* represents area of mologram or cell in μm^2^, η_*A*_ is the analyte efficiency.

**Table 1:** Summary of Estimated RGD-Associated Integrin-Equivalent Densities during 2 h of Cell Adhesion Derived from CMD and Corrected for the Analyte Efficiency

phase	CMD (pg/mm^2^)	integrin equivalent molecules/μm^2^	total integrin equivalent molecules per mologram (160,000 μm^2^)	total integrin equivalent molecules per cell (600 μm^2^)
**I**	75	1810	2.89 × 10^8^	1.08 × 10^6^
**II**	10	241	3.86 × 10^7^	1.45 × 10^5^
**III**	–	–	**3.28 × 10** ^**8**^ (I + II)	**1.22 × 10** ^**6**^ (I + II)

In Phase II, an additional CMD contribution of ∼10 pg/mm^2^ associated with groove-region engagement corresponded to an estimated integrin-equivalent density of 241 molecules/μm^2^, yielding 3.86 × 10^7^ integrin-equivalent molecules per mologram and 1.45 × 10^5^ molecules per cell (corrected for the analyte efficiency). During Phase III, increasing spatial organization and reclustering of adhesion-associated integrin assemblies toward regions of higher diffraction efficiency resulted in a cumulative estimated density of 3.28 × 10^8^ RGD-specific integrin-equivalent molecules on the mologram surface, corresponding to 1.22 × 10^6^ molecules per cell after 2 h of adhesion (Table 1).

These values are in good agreement with literature-reported integrin expression levels. For example, the estimated integrin levels in endothelial cells are (2.1 ± 0.2) × 10^5^ for αVβ3, (2.3 ± 0.2) × 10^5^ for α5β1, and (1.6 ± 0.2) × 10^5^ for αVβ5.^46^ In HeLa cells, the total number of RGD-specific integrins has been estimated to be ∼6 × 10^5^ per cell,^13^ while the αV (CD51) level was measured at ∼2.13 × 10^5^ molecules per HeLa cell.^47^ It is important to note that the integrin expression level can vary depending on multiple factors, such as the activation state of integrins, ECM coating type, and cell density. While the total number of RGD-binding integrins typically falls within the 10^5^–10^6^ range per adherent HeLa cell, only a fraction adopts the high-affinity (active) conformation during cell adhesion. As a result, the number of active RGD receptors is generally estimated to lie within the 10^4^–10^5^ range per cell.^48^

Based on the sensorgram data, and our above hypothesis, we can estimate an apparent integrin nanoscale redistribution rate of integrin-associated adhesion assemblies from grooves toward ridge regions. During Phase III, approximately 13% of the coherently surface-bound integrins appeared to reorganize over a distance of 200 nm within 130 min, corresponding to an effective redistribution velocity of approximately 0.09 μm/h. However, this value should be interpreted with caution. In the early stages of adhesion and spreading, different adhesion structures–nascent adhesions, focal complexes, and focal adhesions–coexist and mature in parallel. Thus, the biosensor signal likely reflects not only lateral redistribution of integrin-associated assemblies but also adhesion complex growth, molecular recruitment, nanoscale remodeling, and spatial reorganization at the cell–surface interface.

Integrins move laterally within the cell membrane via two principal mechanisms: passive diffusion and directed, actomyosin-driven translocation. In their unbound state, they diffuse freely, with α5β1 integrins exhibiting diffusion coefficients ranging from 1.3 to 20 × 10^–10^ cm^2^/s.^49^ Upon ligand engagement and cytoskeletal anchoring, integrins either immobilize or translocate centripetally, as observed by Ballestrem et al. in migrating cells.^50^ Ligand affinity further modulates this mobility: high-affinity integrins cluster more readily and exhibit ∼50% reduced diffusion.^51^

In our experiment, the estimated effective redistribution velocity (0.09 μm/h) is substantially lower than literature values such as 6.5 μm/h reported by Pankov et al. for fibroblasts^52^ (see Supporting Information, Table S1). In contrast, Root Mean Squared (RMS) displacements derived from typical diffusion coefficients suggest that diffusive motion allows integrins to explore areas up to ∼10–30 μm per hour–2 orders of magnitude larger than what directed motion achieves under our conditions (see Supporting Information, Table S2). This highlights diffusion as the dominant lateral transport mechanism in our setup, while directed translocation appears to operate more locally. In addition, transient force-dependent integrin unbinding and adhesion turnover events during early focal adhesion maturation may further contribute to the relatively slow net redistribution observed in the CMD signal. The observed relatively low rate in our system might be in connection with the fact that integrins can also bind to grooves with lower RGD density, slowing down their effective redistribution to higher RGD density ridges with presumably more stable integrin clusters. Therefore, the effective redistribution dynamics are likely governed by a complex interplay between ligand availability, integrin cluster stability, cytoskeletal coupling, and force-dependent adhesion remodeling. Please note that the data obtained in this study reflect the early phase of cell adhesion (within the first 2 h), whereas most literature data are derived from mature adhesions–often in fully spread or even migrating cells. In addition, in a crowded cellular environment, integrin translocation speed can be significantly reduced compared to sparse cell conditions. Dense cell packing creates physical barriers that restrict integrin lateral diffusion in the membrane. In crowded conditions, neighboring cells may additionally act as compliant mechanical buffers that partially absorb cellular contractile forces, thereby reducing the effective tension transmitted to the RGD-functionalized substrate and slowing focal adhesion maturation dynamics. The diffusion of integrins during initial cell adhesion can also be influenced by the presence or absence of serum in the culture medium, with serum-containing conditions creating a complex competitive environment that fundamentally alters integrin mobility and adhesive dynamics.

Simultaneous conventional optical microscopy during FM measurements is currently limited primarily by the instrument configuration. The FM setup requires highly controlled coherent illumination and precise alignment of the diffraction-based detection geometry, which restricted the integration of an additional high-resolution live-cell microscopy path during signal acquisition. Future correlative experiments directly visualizing integrin dynamics, such as live-cell TIRF microscopy, super-resolution imaging, single-particle tracking, or fluorescently tagged integrin knock-ins, will therefore be essential to disentangle the relative contributions of adhesion complex growth, molecular recruitment, and true lateral integrin redistribution. Super-resolution approaches, particularly single-molecule localization methods like PALM and STORM, have already been transformative in unraveling integrin dynamics at the nanoscale in live cells. These approaches provide unprecedented spatial resolution, enabling visualization of individual integrin molecules and their clustering behavior. However, their use is constrained by limitations such as phototoxicity, photobleaching, and long acquisition times, which generally restrict continuous single-molecule tracking to durations of only seconds to minutes. Moreover, nanopatterned substrates themselves profoundly shape integrin behavior by acting as strong attractors, concentrating integrins into dense nanoclusters and “islands”. This organization is likely influenced by complex mechanotransduction pathways, sequential molecular interactions, and the reinforcement of integrin–cytoskeleton linkages in response to mechanical cues, underscoring the central role of nanoscale topography in regulating adhesion and migration. Accordingly, FM-derived redistribution dynamics should be interpreted as integrated structural readouts of adhesion remodeling processes occurring at the cell–surface interface rather than direct measurements of isolated integrin transport behavior.

### Cell Adhesion Kinetics at Different Cell Densities to RGD Motifs Measured by FM

We assessed the kinetics of HeLa cell adhesion to RGD-functionalized surfaces using FM, focusing on the effect of varying cell densities. Figure 5 presents the CMD, RMD and RIU sensorgrams over time for three different cell concentrations (4000, 8000, and 20,000 cells). Upon cell seeding, a rapid increase in CMD was observed, with the magnitude of this increase directly correlating with the number of cells introduced. The highest cell density (Figure 5c) produced the most pronounced adhesion response, reaching a maximum CMD of approximately 57 pg/mm^2^ within 15 min. It is noteworthy that the adhesion kinetics of 20,000 cells are similar to those shown in Figure 3a for 30,000 cells, as both result in densely packed surfaces. The intermediate (Figure 5b) and lowest (Figure 5a) densities resulted in lower maximum values–around 30 pg/mm^2^ and 20 pg/mm^2^, respectively–indicating a clear, cell number-dependent increase in initial attachment.

**Figure 5:**
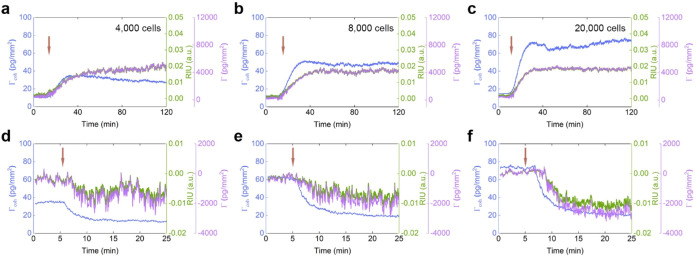
HeLa cell adhesion at varying concentrations to RGD-motifs. Mologram signals (CMD – blue, RIU – green, and RMD – purple) following the addition of (a) 4000, (b) 8000, and (c) 20,000 cells. Cell detachment induced by EDTA is shown for (d) 4000, (e) 8000, and (f) 20,000 cells in a single channel of MoloChip (7.29 mm^2^). Arrows indicate the time points of cell addition (a–c) or EDTA introduction (d–f).

HeLa cell density significantly influences adhesion kinetics and the spatial organization of adhesion-associated signals. At lower concentrations, fewer cells interact with the RGD motifs, resulting in a reduced overall adhesion signal (Figure 5a). Interestingly, the CMD signal decreases over time, while the RIU and the RMD signals increase (Figure 5a). Because the substrate is freely accessible and ligand density is high, HeLa cells in sparse conditions can spread extensively and form large adhesion structures along RGD-rich ridges, forming anisotropic stress fibers aligned along the ridges.^53,54^ In sparse conditions, HeLa cells generate large protrusions and demonstrate increased motility.^55^ The increase in RIU and RMD signals reflects mass accumulation due to cytoskeletal engagement, membrane extension, and the recruitment of adhesion-related proteins to the basal interface. The transient decrease in CMD is consistent with nanoscale redistribution of integrin-associated adhesion material toward previously under-occupied regions of the nanopattern (e.g., groove regions), which reduces the spatial modulation of the refractive index (backfilling effect) and thereby decreases diffraction contrast. This minimizes the refractive index differences between ridges and grooves, further diminishing the contrast essential for coherent mass density detection. Importantly, focal adhesion maturation is fundamentally mechanosensitive and strongly dependent on actomyosin-generated tension rather than being driven solely by spatial or density-related effects. While cell density modulates the mechanical environment, it does not act as a primary determinant of focal adhesion maturation.

The theoretical maximum number of cells that can adhere to a given surface can be estimated using the concept of Random Sequential Adsorption (RSA), which models the irreversible, random placement of particles on a surface without overlap or rearrangement. Under RSA conditions, the jamming limit defines the maximum packing density achievable through such a process. For disk-like objects in two dimensions, this limit is approximately 54.7% of the total available area. In the case of HeLa cells with an average projected area of 300 μm^2^, seeded onto a 160,000 μm^2^ surface (e.g., a 400 μm × 400 μm mologram), the RSA jamming model predicts a theoretical maximum of 292 cells. This estimate assumes cells behave approximately as circular disks during initial contact with the surface. While real cell shapes can vary and may not be perfectly circular, this approximation provides a useful upper bound for monolayer coverage under nonoverlapping, random seeding conditions. HeLa cells, however, lack contact inhibition of growth. In HeLa cultures exceeding 100% confluence, physical formation of FAs is likely limited, resulting in smaller and fewer FAs per cell. Cells located in additional multilayers do not contribute to the specific integrin–RGD-captured signal; however, they may still generate integrin-mediated signals during intracellular signaling processes. In a crowded environment, HeLa cells utilize N-cadherin-mediated adhesion,^56^ and through the actin cytoskeleton, cadherins and integrins cooperate to form an interdependent functional network that translates mechanical inputs into intracellular signals.^55^ N-cadherin adhesion helps limit cell protrusions at cell–cell contacts, promoting coordinated movement of the cell cluster in a single direction.^57^

HeLa cells exhibit distinct adhesion complex dynamics within the first 2 h of adhesion, with sparse and crowded conditions driving divergent mechanobiological responses. In sparse cultures, cells spread extensively and form larger focal adhesion structures (1–3 μm^2^), associated with integrin–ECM engagement, actin stress fiber anchoring, and force-dependent recruitment of vinculin, talin, and FAK, facilitated by kindlin-2 phase separation and oligomerization.^58,59^ In crowded environments, however, focal adhesion maturation is modulated not only by spatial confinement but also by altered mechanical coupling between cells and the substrate. As neighboring HeLa cells begin to establish contact within the first 2 h, adjacent cells act as compliant mechanical buffers that partially redistribute contractile forces. Consequently, part of the actomyosin-generated tension is redirected toward neighboring cells rather than transmitted exclusively to the rigid RGD-functionalized substrate. This mechanical shielding effect reduces the local tension required for focal adhesion maturation, resulting in smaller and more peripheral adhesions (0.3–1 μm^2^), fragmented actin organization, and reduced adhesion stability.^56,60^ Increased cell–cell proximity may additionally interfere with integrin signaling and suppress RhoA-driven contractility. In this context, FM does not directly measure traction force generation, but rather detects changes in the collective nanoscale organization and spatial redistribution of integrin-associated adhesion assemblies during adhesion maturation. This interpretation is consistent with previous studies demonstrating a quantitative correlation between the amount of material in the adhesion zone (within the evanescent field) and the adhesion forces of HeLa cells, where RWG readouts were calibrated against robotic FluidFM single-cell force spectroscopy measurements.^61^ Therefore, the measured CMD should be interpreted as a structural organization parameter emerging from the coupled interplay between ligand density, cytoskeletal tension, integrin clustering, and cell–cell mechanical interactions.

After 120 min of cell adhesion, the channels were washed with DPBS to remove nonadherent cells. The baseline was then recorded in DPBS, and 10 μL of 10 mM EDTA was added to the channels to detach the adhered cells (Figure 5d–f). Please note that enzymatic solutions should be avoided for cell removal, as they may degrade the biomimetic surface. EDTA causes cell detachment from the RGD-coated surface by inactivating integrin-mediated adhesion through chelation of essential divalent cations. A decreasing signal indicates successful cell detachment. Afterward, the chip was rinsed several times with DPBS and reused in repeated measurements.

From the microscopy images, each HeLa cell covers an average surface area of approximately 600 μm^2^ on the mologram’s surface. Supposedly, approximately 12,150 HeLa cells can fit on the 8.1 mm × 0.9 mm (7.29 mm^2^) surface of one channel of MoloChip. However, in practice, to achieve optimal and uniform surface coverage, it is often necessary to seed more cells than the theoretical monolayer maximum. This is because not all cells will attach immediately or evenly, may experience weak adhesion, or fail to settle near adhesion-promoting regions. Therefore, overseeding is a common strategy to ensure that the entire channel becomes fully occupied by spread cells over time. Seeding cells at low concentrations can lead to nonuniform distribution, where cells accumulate unevenly across different molograms within the same channel. Such heterogeneity in cell density can result in signal deviations between molograms, potentially affecting assay reproducibility and interpretation, especially in surface-sensitive optical biosensing applications. Assuming an even distribution and a cell diameter of ∼27.64 μm, we calculated surface coverage density and estimated average cell distances. These metrics were evaluated after seeding (Table 2) and after 2 h of adhesion (Table 3) on RGD-functionalized surfaces: the 7.29 mm^2^ MoloChip channel and the 5.6 mm^2^ RWG sensor well of a 384-well microplate.

**Table 2:** Estimated Initial Spacing and Coverage for HeLa Cells (300 μm^2^) Seeded on the One MoloChip Channel (7.29 mm^2^) and One RWG Sensor Well (5.6 mm^2^) Surfaces

surface area (mm^2^)	cells	cells/mm^2^	theoretical confluence (%)	area per cell (μm^2^)	center-to-center spacing (μm)	edge-to-edge distance (μm)	notes
7.29	4000	549	16.5	1822.5	42.7	23.2	sparse
7.29	8000	1097	33.0	911.25	30.2	10.7	moderate spacing
7.29	20,000	2743	82.3	364.5	19.1	–	near confluent/overlap
5.6	5000	893	26.8	1120	33.5	13.9	moderate spacing
5.6	10,000	1786	53.6	560	23.7	4.2	near contact
5.6	21,000	3750	>100	266.7	16.3	–	overlapping
5.6	42,000	7500	>100	133.3	11.5	–	strong overlap/multilayers

**Table 3:** Estimated Spacing and Coverage for Spread HeLa Cells (600 μm^2^) on the One MoloChip Channel (7.29 mm^2^) and One RWG Sensor Well (5.6 mm^2^) Surfaces after 2 h of Adhesion

surface area (mm^2^)	cells	cells/mm^2^	theoretical confluence (%)	area per cell (μm^2^)	center-to-center spacing (μm)	edge-to-edge distance (μm)	notes
7.29	4000	549	32.9	1822.5	42.7	15.1	moderate spacing
7.29	8000	1097	65.9	911.3	30.2	2.6	near contact
7.29	20,000	2743	>100	364.5	19.1	–	strong overlap
5.6	5000	893	53.6	1120	33.5	5.9	loose
5.6	10,000	1786	107.1	560	23.7	–	crowding starts
5.6	21,000	3750	>100	266.7	16.3	–	strong overlap
5.6	42,000	7500	>100	133.3	11.5	–	strong overlap/multilayers

### Focal Molography Reveals Effects of Glycocalyx Digestion and Histamine-Induced Signaling on Cell Adhesion

In HeLa cells and other cancer types, overexpression of bulky glycoproteins leads to a thickened glycocalyx, which can paradoxically enhance cell adhesion despite increasing the physical distance between the cell membrane and the ECM. This effect has been attributed to the glycocalyx-induced clustering of integrins. Integrin clustering promotes cooperative binding and results in stronger adhesion than what would be achieved by diffusely distributed receptors. Interestingly, enzymatic removal or reduction of different glycocalyx components can lead to the opposite outcomes in cell adhesion.^9^

Neuraminidase is an enzyme that cleaves sialic acid residues from glycoproteins on the cell surface. Sialic acid residues are negatively charged carbohydrates that are found in the oligosaccharide chains of many glycoproteins and glycolipids on the cell surface. The removal of negatively charged sialic acids alters the overall charge distribution on the cell surface. This change in the electrostatic environment may indirectly affect the binding affinity of integrins to RGD peptides.

Sialic acids play a crucial role in maintaining the proper conformation and activation state of integrins. Removal of sialic acid by neuraminidase treatment may alter the activation state of integrins, potentially reducing their ability to bind RGD motifs effectively. Sialic acids contribute to the lateral mobility and clustering of integrins on the cell surface.^62^ Neuraminidase treatment may disrupt this organization, affecting the avidity of integrin-RGD interactions.

To ensure optimal enzymatic activity and avoid interference from serum proteins, the cell adhesion experiments were conducted in buffer (20 mM HEPES in HBSS) instead of standard cell culture medium (Figure 6a,b). Following cell addition, control HeLa cells showed a rapid and robust increase in CMD (Figure 6a, blue), consistent with efficient integrin-mediated adhesion to the RGD-coated ridges of the nanopatterned MoloChip surface.

**Figure 6:**
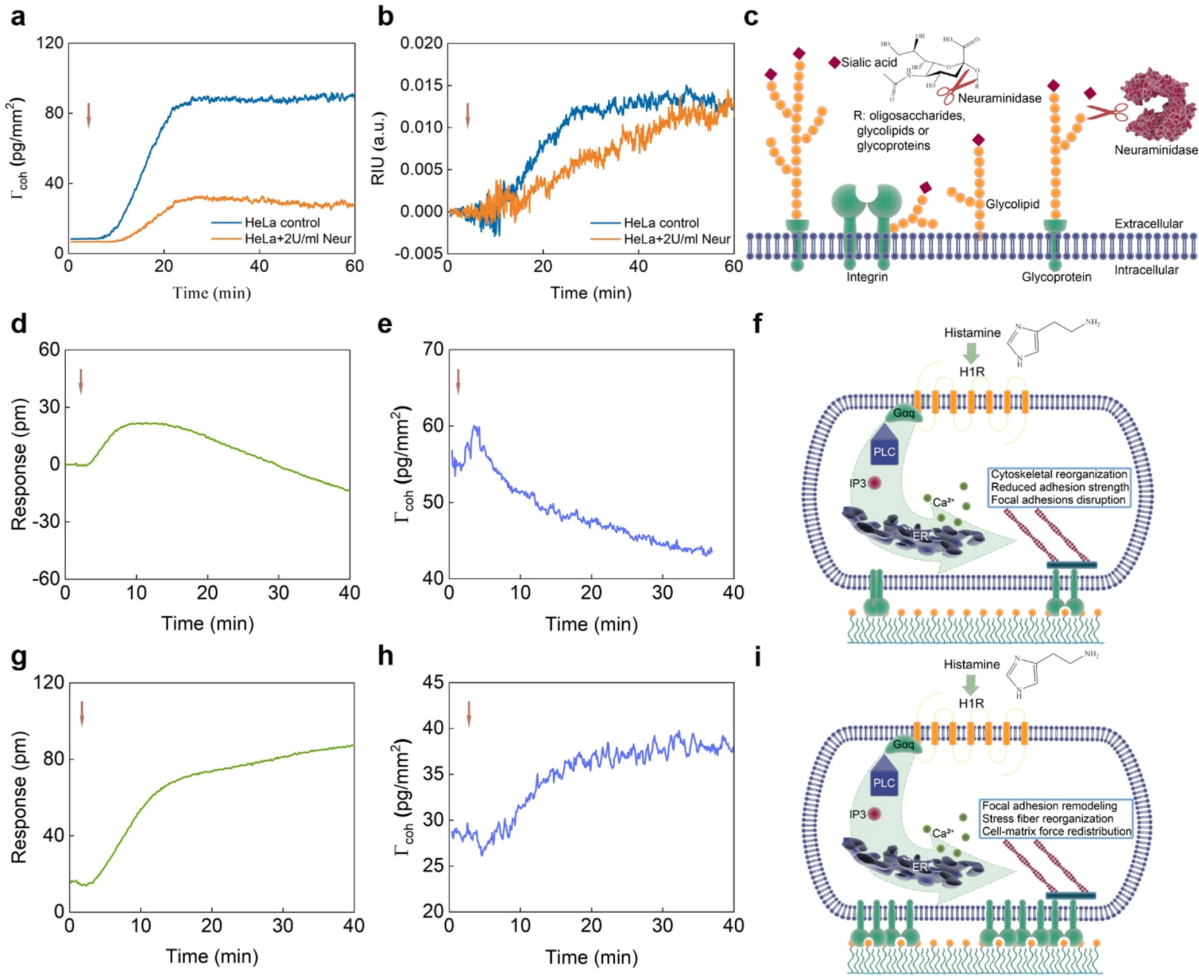
Modulation of HeLa cell adhesion and signaling via neuraminidase treatment and histamine stimulation. (a) CMD changes over time, indicating the amount of specifically bound cellular material. (b) RIU over 60 min following the addition of untreated (blue) and neuraminidase-treated (orange) HeLa cells. (c) Schematic illustration of sialic acid cleavage by neuraminidase. (d–i) HeLa cell response to histamine in DMEM after adhesion to RGD motifs. After 2 h of adhesion, responses were recorded by (d) RWG (3750 cells/mm^2^) and (e) FM (4115 cells/mm^2^). Histamine-induced signals after 24 h of adhesion are shown for (g) RWG and (h) FM. Arrows indicate the time points of cell addition (a, b) or histamine stimulation (d, e, g, h). Schematic illustration of histamine action on the cell after 2 h (f) and 24 h (i) of adhesion: histamine induces the G protein-coupled receptor (Gαq)/phospholipase C (PLC)/inositol trisphosphate (IP3)/Ca^2+^/PKC pathway activation and Ca^2+^ release from endoplasmic reticulum (ER), followed by rapid cytoskeleton response through cytoskeletal reorganization and transient weakening (f) or tightening (i) of adhesion depending on prior adhesion state.

In contrast, neuraminidase-treated cells exhibited a markedly reduced and slower adhesion response (Figure 6a, orange). Removal of terminal sialic acids alters the glycosylation pattern of β1-integrins, which has been reported to affect their lateral mobility and distribution, including changes in receptor mobility fractions.^63^ This modification may therefore influence multiple aspects of the early adhesion process, including integrin clustering, conformational activation, and membrane dynamics. Consistently, neuraminidase treatment also resulted in a delayed and attenuated increase in RIU response (Figure 6b, orange). These results collectively demonstrate that enzymatic deglycosylation affects HeLa cell adhesion kinetics and mass accumulation, and that FM sensitively captures these alterations in real time.

HeLa cells’ response to histamine was investigated by measuring changes in cellular behavior using RWG (Figure 6d,g) and FM techniques (Figure 6e,h). Histamine acts on HeLa cells through G-protein-coupled histamine receptors (H1R), triggering intracellular signaling cascades such as calcium mobilization or cytoskeletal rearrangements (Figure 6f,i).

Histamine exerts complex effects on HeLa cells that are modulated by culturing and measurement conditions,^64^ cell density,^64,65^ and surface adhesive motifs like RGD.^66^ Spatial distribution of RGD ligands influences the balance between outside-in and inside-out signaling processes and associated adhesion organization.

The observed kinetics are consistent with these dynamic changes. Upon histamine stimulation, RWG sensors captured rapid wavelength shifts in HeLa cells (Figure 6d,g), reflecting changes in cell–substrate mass redistribution and adhesion-related reorganization. These responses are consistent with H1R activation and downstream signaling cascades that affect cytoskeletal dynamics.

FM reports changes in the collective nanoscale organization of integrin-associated adhesion structures and associated cellular material at the cell–substrate interface, which can be modulated by histamine-induced signaling and cytoskeletal remodeling.

Histamine responses were strongly influenced by adhesion time, with 24 h cultures exhibiting more robust and reproducible RWG (Figure 6g) and FM (Figure 6h) signals compared to 2 h cultures. Longer adhesion periods are associated with more mature adhesion states, characterized by enhanced FA formation, increased concentration of adhesion molecules (e.g., integrins), and a reorganized actin cytoskeleton. These conditions support more consistent receptor signaling dynamics, whereas shorter adhesion times may introduce variability due to immature adhesion structures and unstable cell–substrate contacts. At 2 h, histamine may induce transient changes in adhesion organization, including redistribution of integrin-associated complexes at the RGD-patterned surface, which can be detected as a reduction in coherent signal. H1 receptors activation is known to modulate cytoskeletal contractility and membrane tension, which may lead to partial cell reorganization or reduced effective contact with the sensor surface.

## Conclusions

This study demonstrates that FM, a label-free optical biosensing technique, enables high-resolution, real-time analysis of HeLa cell adhesion to RGD-functionalized surfaces. Our analysis validates the fundamental operating principle of FM: its ability to distinguish between specific, spatially coherent molecular binding and nonspecific, diffuse mass accumulation. The CMD signal selectively amplifies only the mass that adheres to the sinusoidal nanopattern of the mologram, while total RMD captures all material within the evanescent sensing volume. This specificity allows FM to function as a high-fidelity spatial filter for dynamic molecular reorganization.

Unlike fluorescence recovery after photobleaching, which requires photobleaching and yields ensemble-averaged diffusion rates, FM preserves the native state of integrins and can resolve subtle conformational or clustering changes without perturbation. Compared to single-particle tracking, which depends on sparse labeling and is limited to following a small number of molecules, FM measures the collective behavior of integrins within their physiological density range. In contrast to super-resolution fluorescence techniques (e.g., PALM, STORM), which provide nanometer-scale localization but require labels and long acquisition times, FM achieves high temporal resolution and continuous monitoring in a completely label-free manner. This combination of sensitivity, noninvasiveness, and kinetic resolution allows FM to capture early adhesion complex formation, integrin clustering, and conformational transitions under truly physiological conditions.

FM uniquely captures nanoscale organizational dynamics associated with focal adhesion maturation through coherent mass density modulation and assessment of process coherence. This label-free technique reveals three distinct phases of adhesion characterized by unique coherence signatures. Importantly, FM does not directly measure traction forces or single-molecule transport, but instead provides an integrated optical readout of the collective nanoscale organization and spatial coherence of integrin-associated molecular assemblies within the evanescent field. The observed CMD dynamics therefore reflect coupled processes including integrin clustering, adhesion remodeling, cytoskeletal tension, and force-dependent structural reorganization during focal adhesion maturation. Additionally, cell density modulated adhesion kinetics with sparse cultures favoring larger and more stable adhesions compared to confluent layers, likely due to altered mechanical coupling between cells and the substrate.

Enzymatic removal of sialic acids from the glycocalyx using neuraminidase significantly attenuated adhesion, highlighting the crucial role of integrin clustering and membrane organization in establishing stable cell–substrate interactions. These findings demonstrate that enzymatic deglycosylation alters adhesion kinetics and mass accumulation, and that FM can sensitively detect these changes in real time.

Furthermore, histamine stimulation triggered rapid cytoskeleton-associated remodeling and adhesion-related signaling events, reflecting dynamic changes in cell–substrate interactions. These responses depended strongly on the duration of prior cell adhesion, likely reflecting progressive maturation of FAs, increased integrin clustering, and reinforcement of actomyosin contractility in longer-adhered cells. Since the histamine H1 receptor belongs to the G protein-coupled receptor superfamily and plays important roles in adhesion, migration, inflammatory signaling, and barrier regulation, these findings further highlight the potential of FM for investigating dynamic receptor-mediated mechanobiological processes and pharmacological responses in living cells.

Collectively, these results provide new insights into how extracellular matrix cues, glycocalyx composition, cytoskeletal tension, and intracellular signaling pathways jointly regulate HeLa cell adhesion and adhesion-associated nanoscale organization. They underscore the value of advanced label-free biosensors like FM for investigating complex and dynamic cell–surface interactions.

The FM method is broadly applicable to a wide range of both cancerous and noncancerous adherent cell types, as it detects the collective nanoscale organization and redistribution of receptor-associated molecular assemblies near the cell–substrate interface. Beyond HeLa cells, FM can provide valuable real-time, label-free information on integrin clustering dynamics, adhesion maturation, cell spreading behavior, receptor-mediated signaling, and cytoskeleton-coupled adhesion remodeling. Because the CMD signal reflects structural organization and spatial coherence rather than only total accumulated mass, the method can generate characteristic adhesion “fingerprints” associated with distinct cellular phenotypes and mechanobiological states. In noncancerous cells, FM may be particularly useful for studying physiological adhesion regulation, differentiation, immune-cell interactions, and tissue-engineering related cell–material interactions. In cancer cells, the method could help characterize altered adhesion dynamics, focal adhesion turnover, and mechanobiological phenotypes associated with invasiveness, metastatic potential, and therapy resistance. Furthermore, FM represents a promising platform for mechano-pharmacological studies by enabling label-free monitoring of how drugs targeting cytoskeletal contractility, integrin binding, focal adhesion stability, or mechanotransduction pathways dynamically alter nanoscale adhesion organization, even in complex cellular environments. Beyond fundamental studies of cell adhesion, FM may also provide a valuable approach for investigating therapy-induced mechanobiological remodeling in cancer cells. Since chemotherapy and radiation frequently induce cellular senescence accompanied by profound changes in focal adhesion organization, cytoskeletal tension, and integrin signaling, FM could enable continuous real-time monitoring of these dynamic phenotypic transitions. The method may therefore prove useful for identifying persistent senescent cancer cell populations, studying recurrence-associated adhesion phenotypes, and evaluating senolytic or antimetastatic treatment strategies.

## Methods

### Chemicals

All chemicals and reagents were obtained from Merck KGaA (Darmstadt, Germany), unless stated otherwise.

### Holographic Microscopy

The morphological characteristics of HeLa cells during adhesion were analyzed using the HoloMonitor M4, a label-free, time-lapse cytometer (Phase Holographic Imaging AB, Lund, Sweden). This noninvasive imaging system enables real-time monitoring of live cells under standard culture conditions (37 °C, 5% CO_2_). Three-dimensional cell structures were visualized by illuminating the sample with a 0.1 mW/cm^2^ HeNe laser (635 nm). Holographic images were generated by capturing the interference pattern between reference and object beams on a digital sensor, allowing high-resolution, label-free analysis of cell morphology and behavior. Images were acquired every 5 min inside a humidified incubator using the HoloStudio M4 software.

### Resonant Waveguide Grating Biosensor (RWG)

The RWG cell adhesion assays were performed using a 384-well sensor microplate (#5040, Corning Incorporated, Corning, NY, USA) interrogated by an Epic BT instrument (Corning Incorporated, Corning, NY, USA). Each well of the microplate contains an individual RWG biosensor with a 2 × 2 mm^2^ sensing area of a Nb_2_O_5_ waveguiding layer on a corrugated glass substrate. The corrugation acts as a grating, coupling incident light into the waveguide and generating a waveguide mode with an evanescent field (150 nm penetration depth). The resonant wavelength, where this field is created, shifts when the local refractive index changes. This shift is tracked in real-time to monitor adhesion events and kinetics. The microplate enables 384 parallel measurements, with wavelength scanning (825–840 nm) every 3 s, high-speed imaging via a complementary metal-oxide semiconductor (CMOS) camera, and a spatial resolution of 80 μm. The working principles of the biosensor instrument can be found in refs 12,67 .

### Focal Molography (FM)

FM measurements were performed using the prototype MoloReader ’Callisto Generation’ instrument (lino Biotech AG, Adliswil, ZH, Switzerland) operating with a readout wavelength of 785 nm. The system utilized MoloChip [Me-Tz|PEG] biosensors (lino Biotech AG, Adliswil, ZH, Switzerland), which were placed into a four-channel open cuvette holder for the experiments. Each channel of the cuvette has a usable volume of 18 μL and contains nine square mologram spots (400 μm × 400 μm), providing approximately 9 × 10^10^ binding sites per channel.

The experiments were conducted at room temperature. All sample additions were performed manually, as no autosampler was used. Data points representing the coherent surface mass density (Γ_coh_) were acquired every 2.8 s.

### Preparation of the Biosensor Surfaces with Biomimetic Coating for Cell Adhesion Studies

#### RWG Sensor Surface Preparation

The stock solution of 1.0 mg/mL of the synthetic polymer, PLL(20)-*g*[3.5]- PEG­(2)/PEG­(3.5)-(DBCO-Mal)-CKK-(Acp)­3-GRGDS (PPR, SuSoS AG Dübendorf, Switzerland) was prepared in 10 mM 4-(2-Hydroxyethyl)­piperazine-1-ethanesulfonic acid (HEPES) buffer, pH 7.4, and sterile-filtered. For surface coating, 30 μL of this solution was added to the prewetted wells of RWG biosensor plate and incubated for 30 min at room temperature on a shaking machine. Reagent excess was removed by rinsing the surface with 10 mM HEPES buffer and then the wells were filled with 20 μL of DMEM.

#### Moloreader Chip Surface Preparation

The chip holder and sealing components were cleaned by immersion in 1% Cobas Integra cleaner for 10 min, followed by thorough rinsing with Milli-Q water. Components were then sonicated twice for 5 min each, rinsed again with water, and dried using a stream of pressurized nitrogen.

MoloChip 2D PEG-[Me-Tz|PEG] chips were used for all measurements. For surface functionalization via click chemistry, a 100 μM stock solution of the synthetic polymer (TCO-PEG_4_-Mal)-CKK-(Acp)_3_-GRGDS 99% (TCO-RGD; LifeTein, LLC., USA) was prepared in PBS-T (0.01 M phosphate-buffered saline with 0.05% Tween 20).

Channel 1 of the chip contains calibration holograms for waveguide damping estimation and remains uncoated. Functionalization of the working channels (channels 2, 3, or 4) was performed either inside or outside the instrument.

Inside the instrument: The selected working channel was filled with 18 μL of PBS-T, and a baseline signal was recorded for 15 min. PBS-T was then replaced with 100 μM TCO-RGD solution and incubated for 10 min. Unbound reagents were washed out with PBS-T, followed by a final buffer exchange with either 100 μL of DMEM or assay buffer (20 mM HEPES in Hank’s balanced salt solution (HBSS), pH 7.4; referred to as HEPES HBSS buffer).

Outside the instrument: 18 μL of 100 μM TCO-RGD solution was added to the working channel and incubated for 10 min. Excess reagent was removed through repeated washing with PBS-T. The channel was then filled with either DMEM or assay buffer prior to measurement.

### Cell Culture

HeLa cervical cancer cells (#9302113 ECACC) were maintained in Dulbecco’s modified Eagle’s medium (DMEM, high glucose, GlutaMAX Supplement, pyruvate, #31966021 Gibco) supplemented with 10% fetal bovine serum (#A5670701 Gibco), Antibiotic-Antimycotic solution (#15240096 Gibco). Cells were cultured in a humidified atmosphere containing 5% CO_2_ at 37 °C.

For the experiments, cells were removed from tissue culture dishes using 0.05% (w/v) trypsin and 0.02% (w/v) EDTA. The harvested cells were centrifuged at 200*g* for 5 min and the cell pellet was resuspended in DMEM or in assay buffer (20 mM HEPES HBSS buffer). When cell suspension was prepared in HEPES HBSS buffer, the centrifugation was repeated two times to completely remove the cell culture media.

### Cell Adhesion Measurements

#### Cell Adhesion Assay Using HoloMonitor M4

An Ibidi μ-Slide I (hydrophobic, uncoated, Ibidi) was precoated with RGD motifs. Briefly, 120 μL of 1 mg/mL PPR was pipetted into the chamber and incubated for 30 min. Reagent excess was removed by rinsing the surface with 10 mM HEPES buffer, and the channel was then filled with DMEM. Cells were plated at a density of 100,000 cells per channel (2.5 cm^2^).

#### Cell Adhesion Assay Using RWG

Prior to addition of cell suspensions, a baseline was recorded in the sensor wells of the RWG microplate for 30 min with 20 μL of complete DMEM. After the stable baselines had been established for all wells, 20 μL of HeLa cell suspensions (at a concentration of 5000, 10,000, 21,000 and 42,000 cells per 20 μL) were seeded into the wells and the biosensor responses were recorded for 2 h at room temperature.

#### Cell Adhesion Assay Using FM

After buffer exchange, the chip was placed into the instrument, and baselines were established in complete DMEM or 20 mM HEPES HBSS buffer. Ten μL of HeLa cell suspension at different concentrations were added to appropriate channels of open cuvette. Cell adhesion was monitored at room temperature.

#### Quantitative Analysis of HeLa Cells Adhesion Dynamics Using FM

To describe the temporal evolution of adhesion dynamics, both RMD and CMD signals were fitted using phenomenological models that ensured smooth and continuous derivatives suitable for quantitative phase identification.

For RMD, a 4-parameter logistic (4PL) model was employed, extended with a linear drift term to account for slow baseline shifts. The drift was modulated by a sigmoid window to avoid discontinuities at the onset of the measurement

$$RMD\;\left(t\right)=A+\frac{D-A}{1+e^{-B\times t-C/1000}}+linear\;drift\;\left(t\right)$$

where the drift term is defined as

$$\mathrm{linear}\;\mathrm{drift}\;\left(t\right)=\mathrm{slope}\times \left(t-t_{0}\right)\times \mathrm{sigmoid}\;\left(t,t_{0},\mathrm{width}\right)$$

$$sigmoid\;\left(t,t_{0},width\right)=\frac{1}{1+e^{-t-t_{0}/width}}$$

Here, *A* represents the minimum asymptote or baseline, *D* the maximum asymptote, *B* the Hill slope determining the steepness of the transition, and *C* the inflection point (EC_50_, in seconds). The linear drift term is defined by the slope, which gives the rate of baseline change in pg/mm^2^/s, while *t*_0_ indicates the onset time of the drift and the parameter width specifies the transition width of the sigmoid window used to smoothly apply the drift.

CMD dynamics were captured by a combination of three mathematical terms: an initial 4PL rise, a negative sigmoid representing signal decay, and a positive sigmoid describing partial recovery

$$\mathrm{CMD}\;\left(t\right)=4\mathrm{PL}\;\left(t\right)+{\mathrm{sigmoid}}_{1}\;\left(t\right)+{\mathrm{sigmoid}}_{2}\;\left(t\right)$$

with

$$4\mathrm{PL}\;\left(t\right)=A_{1}+\frac{D_{1}-A_{1}}{1+e^{-B_{1}\times t-C_{1}/1000}}$$

$${\mathrm{sigmoid}}_{1}\;\left(t\right)=\frac{A_{2}}{1+e^{-t-t_{2}/w_{2}}}$$

$${\mathrm{sigmoid}}_{2}\;\left(t\right)=\frac{A_{3}}{1+e^{-t-t_{3}/w_{3}}}$$

In the CMD model, the initial 4PL term is described by *A*_1_ and *D*_1_, representing the minimum and maximum asymptotes, respectively, *B*_1_ for the Hill slope determining steepness, and *C*_1_ for the inflection point (EC_50_, in seconds). The first sigmoid term, which accounts for signal decay, is defined by *A*_2_ (amplitude, negative for decay), *t*_2_ (center time in seconds), and *w*_2_ (transition width). The second sigmoid term, describing partial recovery, is similarly defined by *A*_3_ (amplitude, positive for recovery), *t*_3_ (center time), and *w*_3_ (transition width). Together, these three components allow the CMD signal to be decomposed into interpretable phases corresponding to likely molecular processes at the nanopatterned surface.

All parameters were determined by nonlinear least-squares fitting using Python SciPy. Initial guesses for inflection points and amplitudes were derived from the raw traces.

The final parameter values are summarized in Table 4, including asymptotes (*A*, *D*), slopes (*B*), inflection times (*C*), linear drift slope, and sigmoidal transition parameters (*A*_2_, *t*_2_, *w*_2_ *A*_3_, *t*_3_, *w*_3_).

**Table 4:** Fitted Parameters for RMD and CMD Models^a^

parameter	refractometric mass density	coherent mass density
*A*	–100.00 pg/mm^2^	–0.45 pg/mm^2^
*B*	3.95	6.97
*C*	815.41 s	853.82 s
*D*	6360.25 pg/mm^2^	90.67 pg/mm^2^
linear drift slope	0.03 pg/mm^2^/s	-
*A*_2_	-	–30.00 pg/mm^2^
*t*_2_	-	1322.93 s
*w*_2_	-	405.78 s
*A*_3_	-	14.86 pg/mm^2^
*t*_3_	-	8000.00 s
*w*_3_	-	2512.29 s

a RMD was modeled with a 4PL function with linear drift, and CMD with a 4PL plus two sigmoid transitions. Units are provided in the table.

### Neuraminidase Treatment

HeLa cells were treated with neuraminidase (#N2876, Merck KGaA, Darmstadt, Germany) from *Clostridium perfringens* to remove terminal sialic acid residues. The stock solution of the enzyme (10 U/mL) was prepared in 20 mM HEPES HBSS buffer and stored at −20 °C until use.

Cells were seeded in 20 mM HEPES HBSS assay buffer to maintain enzymatic activity and exclude serum interference. Prior to cell seeding, the system baseline was established. Cells were preincubated with 2 U/mL neuraminidase for 5 min at room temperature. Following this, 10 μL of assay buffer was removed from the system and replaced with 10 μL of the neuraminidase-treated cell suspension.

### Histamine Treatment

To assess cellular response dynamics, adherent HeLa cells were treated with histamine (#J61727.03, Thermo Fisher Scientific Inc.). The stock solution was prepared in Dulbecco’s Phosphate-Buffered Saline (DPBS).

For the RWG experiment, cells were allowed to adhere to the RGD-functionalized surface for either 2 or 24 h, after which histamine was added to the culture medium at a final concentration of 50 μM.

For the FM experiment, cells were seeded onto the RGD surface and incubated for either 2 or 24 h. Nonadherent cells were removed by washing, and the adhered cells were subsequently treated with histamine at a final concentration of 25 μM in the medium.

### Microscopy

Microscopic imaging was used to confirm cell adhesion and morphology. In all experiments, phase-contrast imaging was performed after biosensor measurements. Cells were visualized on a sensor chip using a Zeiss Axio Observer.Z1 microscope (Carl Zeiss AG, Oberkochen, Germany) with a 10× objective (421041–9910–000, Carl Zeiss AG).

HeLa cells tagged with Vinculin Monoclonal Antibody (7F9), Alexa Fluor 488 (eBioscience, Thermo Fisher, Waltham, MA, USA) were imaged using a 20× objective and the 38 HE fluorescent filter set (excitation: BP 470/40 nm, emission: BP 525/50 nm).

Both phase contrast and fluorescent images were captured and analyzed using the Zen Blue software (Carl Zeiss AG), and further image processing and analysis were performed using FIJI (ImageJ v2).^68^

## Supplementary Material



## Data Availability

All data needed to evaluate the conclusions in the paper are present in the paper and/or the Supporting Information. Additional data related to this paper may be requested from the corresponding authors.

## Notes

Experimental data on Molecular Rearrangement of Proprietary IgG on [anti-Fc|PEG] Molograms, summary table of integrin diffusion coefficients and translocation speeds under various experimental conditions, and summary table of Root Mean Squared displacement of integrins calculated from reported diffusion coefficients (PDF)
