# Supplementary material for: Organization and Dynamics of Focal Adhesions: Light Diffraction Analysis of Cellular Adhesion on Nanopatterned Surfaces
Source: ACS Appl Mater Interfaces. 2026 Jun 10;18(24):34480–500. doi: 10.1021/acsami.6c04698 (PMC13307071; doi:10.1021/acsami.6c04698)
Supplement: Supplementary file 1 [file am6c04698_si_001.pdf]

Supporting Information

Organization and dynamics of focal adhesions: light diffraction analysis of cellular adhesion on nanopatterned surfaces.

Inna Szekacs[1]*, Szabolcs Novák[1,2], Boglarka Kovacs[1], Zoltán Dicső[1,3], Beatrix Péter[1], Attila Bonyár[2], Roman Popov[4], Andreas Frutiger[4], Robert Horvath[1,5]*

[1]Nanobiosensorics Laboratory, Institute of Technical Physics and Materials Science, Centre for Energy Research, HUN-REN, Konkoly-Thege Miklós Street 29-33, 1121 Budapest, Hungary

[2]Department of Electronics Technology, Faculty of Electrical Engineering and Informatics, Budapest University of Technology and Economics, Egry J. Street 18., 1111 Budapest, Hungary

[3]Department of Biological Physics, ELTE Eötvös Loránd University, 1117 Budapest, Hungary

[4]Lino Biotech AG, Soodstrasse 52, 8134 Adliswil, Switzerland

[5]Institute of Biophysics, Biological Research Centre HUN-REN, 6726 Szeged, Hungary

* **Corresponding authors:** Inna Szekacs (szekacs.inna@ek.hun-ren.hu), Robert Horvath (horvath.robert@ek.hun-ren.hu)

This file contains:

Supplementary Figure S1

Supplementary Table S1

Supplementary Table S2

Molecular Rearrangement of Proprietary IgG on [anti-Fc|PEG] Molograms

To further validate the capacity of focal molography (FM) to detect molecular rearrangement beyond cell surface integrins, we investigated the binding behavior of a proprietary IgG (supplied by lino Biotech AG) on [anti-Fc|PEG] molograms. These surfaces were prepared analogously to the main study via site-specific MeTz-TCO click chemistry, enabling selective immobilization of TCO-activated anti-Fc peptide (a peptide which reversibly bind the Fc domain of the antibody) on the ridges of [Me-Tz|PEG] molograms.

Upon injection of a proprietary IgG (200 nM in phosphate-buffered saline with 0.05% (v/v) Tween-20), the FM sensorgram revealed a rapid increase in both coherent mass density (CMD) and total refractometric mass density (RMD), characteristic of specific binding. Notably, during the association phase, CMD exhibited a gradual decline after reaching its maximum within 2 min post-injection, while RMD remained stable (Fig. S1). This decoupling indicates a redistribution of bound IgG molecules from the densely functionalized ridges into the more sparsely functionalized grooves.

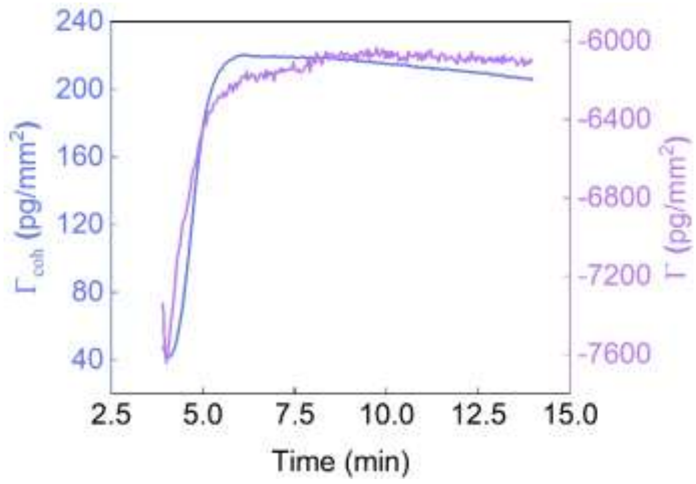

Fig. S1. Coherent and total mass density responses during a proprietary IgG binding on [anti-Fc|PEG] molograms.

Such rearrangement reduces the spatial refractive index modulation across the mologram, thereby decreasing the diffractive efficiency without altering the total surface mass. This mirrors the integrin behavior observed during HeLa cell adhesion, reinforcing the interpretation that focal molography sensitively captures nanoscale molecular dislocation phenomena. These findings affirm FM's utility in detecting lateral reorganization of surface-bound analytes independent of overall mass accumulation.

Table S1. Summary of integrin diffusion coefficients and translocation speeds under various experimental conditions

Study	Integrin type	Diffusion coefficient / Translocation speed	Experimental conditions
Yang et al. (2017)[1]	α5β1	1.4×10^{-10} cm²/s (control)	HeLa cells; single particle tracking (SPT) method
Pankov et al. (2000)[2]	α5β1	Translocation rate: ~6.5 μm/h	Primary human foreskin fibroblasts (HFF) cells; antibody-chasing method, time-lapse fluorescence microscopy
Schootemeijer et al. (1997)[3]	αIIbβ3	3.7×10^{-10} cm²/s	Human megakaryocyte cells; fluorescence recovery after photobleaching (FRAP)
Hirata et al. (2005)[4]	α5β1	Mean: $5.3 \pm 4.4 \times 10^{-10}$ cm²/s	Human fibroblast, non-adhesive, cytoskeleton-free membrane; SPT
Mainali et al. (2012)[5]	αPS2CβPS	1-20 10^{-9} cm²/s	Drosophila S2 cells; SPT and FRAP
Yuan et al. (2022)[6]	β1	3.9-6.3 × 10^{-10} cm²/s	MCF10A, MCF7, MDA-MB-231 cells; SPT
Present work	HeLa-expressed (αvβ3, αvβ5, and α5β1)	~0.09 μm/h (translocation speed)	HeLa cells on RGD-functionalized micropatterned biosensor surface; label-free detection

Table S2. Root Mean Squared (RMS) displacement of integrins calculated from reported diffusion coefficients

Study	Diffusion coefficient (cm^2/s)	RMS displacement (μm) after 1 h
Yang et al. (Gal-3)[1]	1.8×10^{-10}	16.1
Yang et al. (control)[1]	1.4×10^{-10}	14.2
Schootemeijer et al.[3]	3.7×10^{-10}	23.1
Hirata et al.[4] (mean)	5.3×10^{-10}	27.6
Hirata et al.[4] (min)	1.3×10^{-10}	13.7
Hirata et al.[4] (max)	2.0×10^{-9}	53.7
Yuan et al. (MCF10A)[6]	3.9×10^{-10}	23.7
Yuan et al. (MCF10A epithelial to mesenchymal transition induced)[6]	7.0×10^{-10}	31.7
Yuan et al. (MDA-MB-231)[6]	6.3×10^{-10}	30.1
Yuan et al. (MCF7)[6]	4.1×10^{-10}	24.3

References

(1) Yang, E. H.; Rode, J.; Howlader, M. A.; Eckermann, M.; Santos, J. T.; Hernandez Armada, D.; Zheng, R.; Zou, C.; Cairo, C. W. Galectin-3 Alters the Lateral Mobility and Clustering of Β1-Integrin Receptors. *PLoS ONE* **2017**, *12* (10), 1–17. https://doi.org/10.1371/journal.pone.0184378.

(2) Pankov, R.; Cukierman, E.; Katz, B. Z.; Matsumoto, K.; Lin, D. C.; Lin, S.; Hahn, C.; Yamada, K. M. Integrin Dynamics and Matrix Assembly: Tensin-Dependent Translocation of Α5β1 Integrins Promotes Early Fibronectin Fibrillogenesis. *The Journal of Cell Biology* **2000**, *148* (5), 1075. https://doi.org/10.1083/JCB.148.5.1075.

(3) Schootemeijer, A.; van Willigen, G.; van der Vuurst, H.; Tertoolen, L. G. J.; De Laat, S. W.; Akkerman, J.-W. N. Lateral Mobility of Integrin αIIbβ3 (Glycoprotein IIb/IIIa) in the Plasma Membrane of a Human Megakaryocyte. *Thrombosis and Haemostasis* **1997**, *77* (01), 143–149. https://doi.org/10.1055/s-0038-1655922.

(4) Hirata, H.; Ohki, K.; Miyata, H. Mobility of Integrin Α5β1 Measured on the Isolated Ventral Membranes of Human Skin Fibroblasts. *Biochimica et Biophysica Acta (BBA) - General Subjects* **2005**, *1723* (1–3), 100–105. https://doi.org/10.1016/J.BBAGEN.2005.01.014.

(5) Mainali, D.; Smith, E. A. The Effect of Ligand Affinity on Integrins' Lateral Diffusion in Cultured Cells. *European Biophysics Journal* **2013**, *42* (4), 281–290. https://doi.org/10.1007/S00249-012-0873-X/FIGURES/4.

(6) Yuan, J. W.; Zhang, Y. N.; Liu, Y. R.; Li, W.; Dou, S. X.; Wei, Y.; Wang, P. Y.; Li, H. Diffusion Behaviors of Integrins in Single Cells Altered by Epithelial to Mesenchymal Transition. *Small* **2022**, *18* (5), 2106498. https://doi.org/10.1002/SMLL.202106498.
